# Computational challenges in exoplanet atmospheric retrieval

**DOI:** 10.1007/s41115-026-00031-9

**Published:** 2026-08-05

**Authors:** Joanna K. Barstow, Luis Welbanks

**Affiliations:** 1 School of Physical Sciences, The Open University, Walton Hall, Milton Keynes, MK7 6AA UK; 2 School of Earth and Space Exploration, Arizona State University, Interdisciplinary Science & Technology Building IV, 781 Terrace Mall, Tempe, AZ 85287 USA

**Keywords:** Exoplanet atmospheres, Spectroscopy, Astrostatistics techniques

## Abstract

Spectral retrieval is a key computational tool for interpreting spectroscopic observations of exoplanets. As data quality has dramatically improved with the launch of JWST, so the complexity of retrieval models has also increased. Due to the lack of ground truth and prior knowledge for exoplanets, retrievals adopt algorithms capable of exploring a wide parameter space, such as MCMC or Nested Sampling, which become increasingly inefficient for higher dimensionality problems. Tuning retrieval complexity for these scenarios requires a delicate compromise between sufficient physical accuracy and pragmatic approximations. Retrievals can be applied to many different types of observation, with specific challenges associated with each. In the current environment, as we begin characterize smaller, temperate planets that may potentially be habitable, interpretation of retrievals and calculation of detection significance must be approached with special care. In this review, we summarize the current computational challenges in all aspects of the retrieval process: atmospheric modelling; data preparation; inversion and likelihood calculation; and interpretation, for a range of observational strategies. We conclude with a series of recommendations for scientists approaching retrievals for the first time in this new data-rich era for exoplanet science.

## Introduction

Exoplanet spectroscopy has become one of the fastest-growing fields in astronomy over the last twenty years. Going from the photometric studies that first revealed the presence of H$_2$O in the atmosphere of a hot Jupiter (Tinetti et al. 2007) to the exquisitely detailed spectra available from JWST (e.g. Carter et al. 2024) has required a similar step change in the atmospheric models applied to these datasets. Alongside substantial developments in observational capability came a shift in approach, driven in part by an influx of solar system atmospheric scientists joining the field. Spectral retrieval, long a workhorse technique for interpreting remote sensing spectroscopic observations of the Earth and other solar system planets (see e.g. Rodgers 2000; Irwin et al. 2004; Fletcher et al. 2009; Cottini et al. 2012) started to be applied to Hubble Space Telescope transit and eclipse spectra of favourable targets orbiting bright stars (Madhusudhan and Seager 2009; Lee et al. 2012; Line et al. 2013).

Spectral retrieval is a parametric modelling technique that iteratively explores different combinations of parameters and statistically compares the generated synthetic spectra to observational data. Its usefulness lies in the freedom to explore a wide parameter space beyond that encompassed within physically predictive models. Notable retrieval-generated results within solar system science include constraints on the structure of Jupiter’s equatorial cloud decks from the Galileo orbiter (Irwin and Dyudina 2002); retrievals of spatial variation in ammonia on Saturn (Fletcher et al. 2011); and variation in surface emissivity on Venus (Kappel et al. 2016).

Solar system retrievals are typically based on matrix inversion solutions such as Optimal Estimation (Rodgers 2000). Such methods contain implicit assumptions about the Gaussianity of the problem, and in particular require Gaussian (therefore informative) prior constraints. With extensive spectral coverage from multi-instrument orbiters and ground truth from e.g. descent probes, sufficient prior information usually exists to make this type of retrieval viable.

Exoplanet atmosphere study, on the other hand, represents an exploration of the unknown and there is little motivation for informative priors. Early retrieval attempts using Optimal Estimation found that the restrictive Gaussian priors could heavily bias the results of the retrieval (e.g. Barstow et al. 2013), where the solution is shown to be highly dependent on the chosen prior constraint). Line et al. (2013) conduct an investigation into such biases and examine the effect of the choice of retrieval algorithm using synthetic data. They find that the Optimal Estimation algorithm is subject to bias where the data are relatively unconstraining, whereas a Bootstrap Monte Carlo technique is better able to explore the full parameter space and recover the correct solution. The Markov-Chain Monte Carlo (Brooks 1998, MCMC) algorithm was adopted as a standard for exoplanet retrieval (e.g., Benneke and Seager 2012; Waldmann et al. 2015). However, Monte Carlo methods still faced challenges where posteriors were highly multi-modal. An alternative is the Nested Sampling (Skilling 2006) method, which was first trialled by Benneke and Seager (2013). Nested Sampling has the advantages that it is able to represent multi-modal posteriors, and the Bayesian Evidence emerges as a product of the calculation. This facilitates easier model comparison and model selection, which are discussed in more detail in Sect. 6.

There are now in excess of 50 independent retrieval frameworks that have been applied to exoplanets (MacDonald and Batalha 2023). They use a variety of sampling algorithms, temperature and cloud parameterizations, and representations of chemistry from equilibrium/disequilibrium assumptions to completely flexible free retrieval schemes. Many are open-source, and efforts have been made to benchmark some codes to ensure consistency (e.g. Barstow et al. 2020, 2022; Welbanks 2025, the RISOTTO Model Intercomparison Project Sohl et al. 2024). These intercomparisons are required because small differences in model implementation, leading to variation of a few 10 s of ppm in synthetic spectra, can result in sometimes substantial differences in retrieved values (see e.g. Barstow et al. 2020).

A full retrieval analysis for a particular observation or set of observations can often involve multiple retrieval runs, either to test different parameterization approaches or to quantify the evidence for the inclusion of a particular absorber. With more flexible Bayesian samplers, several tens of thousands of individual forward model calculations may need to be calculated. Therefore, a retrieval exploration using an MCMC or Nested Sampling algorithm can be very computationally expensive, and this scales heavily with the speed of the forward model calculation.

This review focuses on retrieval from the perspective of transiting exoplanet observations, noting where different challenges arise for direct imaging and high-resolution cross-correlation observations. While transit spectroscopy currently leads the charge towards population-level studies, the focus is likely to shift in the era of the ELTs and new flagship missions such as NASA’s Habitable Worlds Observatory.

We first explain the basic concept of a retrieval (Sect. 2). We then describe the details of a typical exoplanet atmosphere forward model within the retrieval context (Sect. 3). Many of the computational challenges associated with spectral retrieval arise from introducing successive levels of detail to these models to represent ever-improving observational data (Sect. 4). Section 5 covers the inversion process and how computational treatments of, for example, priors and likelihood can affect results, and Sect. 6 discusses challenges in interpreting retrieval results. Finally, Sect. 7 addresses future challenges as new observatories come online and summarizes our main recommendations.

## Everything you wanted to know about retrievals (but were afraid to ask)

Retrieval is the process of inferring the state of an atmosphere from observational data in a probabilistic way. Regardless of the precise algorithm used, the method of inference is founded on Bayes’ Theorem. Bayes’ Theorem allows us to invert the conditional probability of a measured outcome for a given underlying cause, and determine instead the probability of the underlying cause given the measured outcome — in our case, we can determine the probability of a particular atmospheric configuration given the spectrum we observe. Bayes’ theorem is most simply stated as follows:

1 $$\begin{aligned} P(A \mid B) = \frac{P(B \mid A) P(A)}{P(B)} \,, \end{aligned}$$

where P(A∣B), P(B∣A) are the probabilities of *A* given *B* and vice versa, and *P*(*A*), *P*(*B*) are the probabilities of *A* (the **prior** probability) and *B* (the **marginal** probability).

In the case of atmospheric retrieval, the quantity we are interested in determining is the probability of a set of atmospheric model parameters $\theta _{\mathcal {M}}$ drawn from model $\mathcal {M}$ given the observed data $\mathcal {D}$ (the **posterior** probability distribution). Following Bayes’ theorem, this can be calculated from the **likelihood** $\mathcal {L}=P(\mathcal {D} \mid \theta _{\mathcal {M}},\mathcal {M})$, the probability of observing the data $\mathcal {D}$ given a specific set of model parameters $\theta _{\mathcal {M}}$ drawn from model $\mathcal {M}$; the **prior** $\Pi = P(\theta _{\mathcal {M}}\mid \mathcal {M})$; and the **Bayesian Evidence** for the model $\mathcal {Z} = P(\mathcal {D}\mid \mathcal {M})$:

2 $$\begin{aligned} P(\theta _{\mathcal {M}} \mid \mathcal {D}, \mathcal {M}) = \frac{P(\mathcal {D} \mid \theta _{\mathcal {M}}, \mathcal {M})\, P(\theta _{\mathcal {M}}\mid \mathcal {M})}{P(\mathcal {D}\mid \mathcal {M})} \end{aligned}$$

or

3 $$\begin{aligned} P(\theta _{\mathcal {M}} \mid \mathcal {D}, \mathcal {M}) = \frac{\mathcal {L}\,\Pi }{\mathcal {Z}}. \end{aligned}$$

**Fig. 1:**
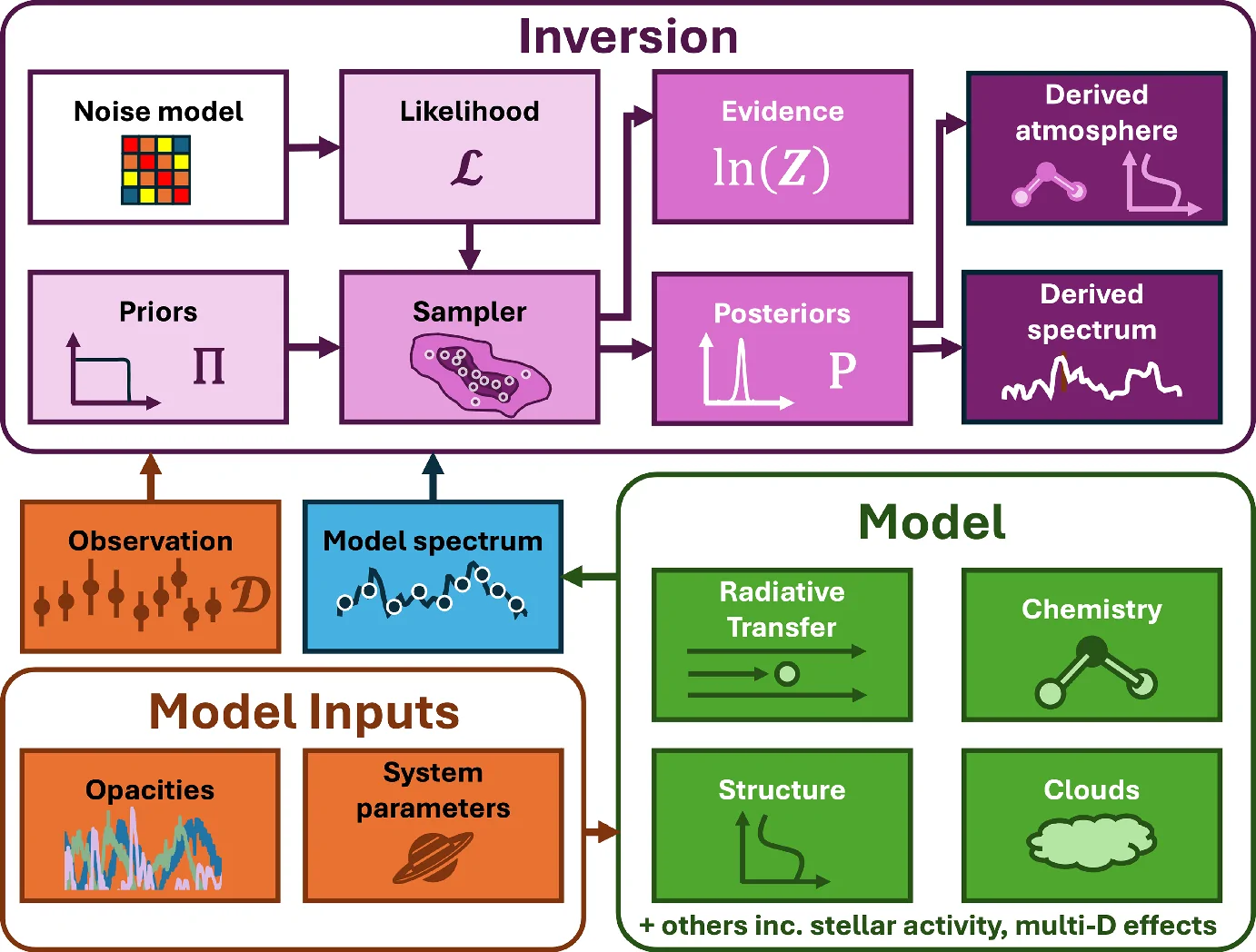
A block diagram of an atmospheric retrieval model. Aspects of the model and inputs will be discussed in Sect. 3. Treatment of data from different sources will be covered in Sect. 4. The inversion process, going from priors to posteriors, will be discussed in Sect. 5. The process of model selection from the retrieval results and subsequent interpretation follows in Sect. 6

The Bayesian Evidence $\mathcal {Z}$ is calculated by integrating the likelihood over the prior volume, so $\mathcal {Z}=\int P(\mathcal {D}\mid \theta _{\mathcal {M}}, \mathcal {M}) P(\theta _{\mathcal {M}}\mid \mathcal {M}) \,d\theta _{\mathcal {M}}$. A comparison of the Bayesian Evidence obtained under different assumptions for model $\mathcal {M}$ can be used to select the most appropriate model (Sect. 6).

The above equations are predicated on the model $\mathcal {M}$ being a suitable representation of the atmosphere, which may not always be the case. We will discuss challenges surrounding this further in Sect. 3. It can also be seen that the choice of the prior Π will have a substantial effect on the outcome of the retrieval.

In Fig. 1 we present a block diagram for the retrieval which shows where each part of Eq. (3) fits into the process. The model $\mathcal {M}$ is constructed based on a radiative transfer calculation, with specified assumptions about atmospheric structure, chemistry and clouds as well as other, more complicated factors. Inputs to the model are the opacities for any gases and aerosols present, and the system parameters for the planet. These aspects are discussed in more detail in Sect. 3. The outcome of this model is a synthetic spectrum, binned to the resolution of the observed spectrum; this together with the observation (data, $\mathcal {D}$) are both fed into the inversion algorithm together with the noise model, which is important for calculating the likelihood $\mathcal {L}$ (discussed further in Sects. 4 and 5). The likelihood for a given model iteration and the priors Π are inputs to the chosen sampler, which on completion provides the Bayesian Evidence $\mathcal {Z}$ and posterior probability distributions as outputs. From the posteriors, we can then construct a best fit spectrum and the corresponding atmosphere state based on the atmospheric model (see Sects. 5 and 6).

## The model

The calculation of the forward model is a key step in the retrieval process, and one that is typically performed many thousands or even millions of times. Therefore, the speed of an individual forward model computation is a key bottleneck for atmospheric retrieval. Forward model set ups differ between codes and according to application, but they generally share the same basic structure. For the most part, they are 1D, although increasingly attempts are being made to encode some (pseudo) 2D, or even 3D, information in these; this will be covered in more detail in Sect. 3.8.2. They include some specification of the atmospheric chemistry, either through explicit linkage to a chemical equilibrium or disequilibrium network, or through independent retrieval of individual gas abundances (see Sect. 3.4). For free chemistry retrievals the atmosphere is often assumed to be well mixed, with gas abundances constant with altitude, but some parameterizations may include variation as a function of altitude. They also include a statement of how temperature varies as a function of pressure (the T-P profile), following one of a range of possible parameterizations from the very simple isothermal atmosphere to a continuous retrieval with some smoothing length.

As well as the representation of the various atmospheric properties within the model, each retrieval code must also incorporate opacity information for each of the gases and aerosols present. This includes opacity for collision-induced absorption for the major atmospheric constituents (e.g. H$_2$ and He for gas giant atmospheres) and for trace species opacity data as a function of wavelength, pressure and temperature are pre-calculated and tabulated in some format (for further details see Sect. 3.5.) For aerosols, the absorption and scattering efficiencies can be parameterized, but they can also be calculated from refractive indices for a given cloud species, assuming a certain size distribution. This is covered further in Sect. 3.6.

### Radiative transfer

At the core of the model is the radiative transfer equation, that quantifies how radiation intensity changes as light passes through an atmosphere with a given composition and structure. The change in intensity is a balance between radiation source and loss terms.

The change in spectral radiance as a function of distance along a path *s* can be written as

4 $$\begin{aligned} \frac{\textrm{d}I_{\lambda }}{\textrm{d}{s}}= -(\alpha _{\lambda }+\sigma _{\lambda })I_{\lambda } + S_{\lambda } \,, \end{aligned}$$

where $I_{\lambda }$ is the spectral radiance at wavelength λ; $\alpha _{\lambda }$ is the absorption coefficient; $\sigma _{\lambda }$ is the scattering coefficient; and $S_{\lambda }$ is a source term that encompasses any radiation intensity emitted or scattered into the beam. The first term on the right hand side represents radiation lost from the beam via absorption or scattering. It can be seen that the absorptive and scattering properties of the medium need to be known in order to solve this equation. This is discussed further in Sects. 3.5 and 3.6.

**Fig. 2:**
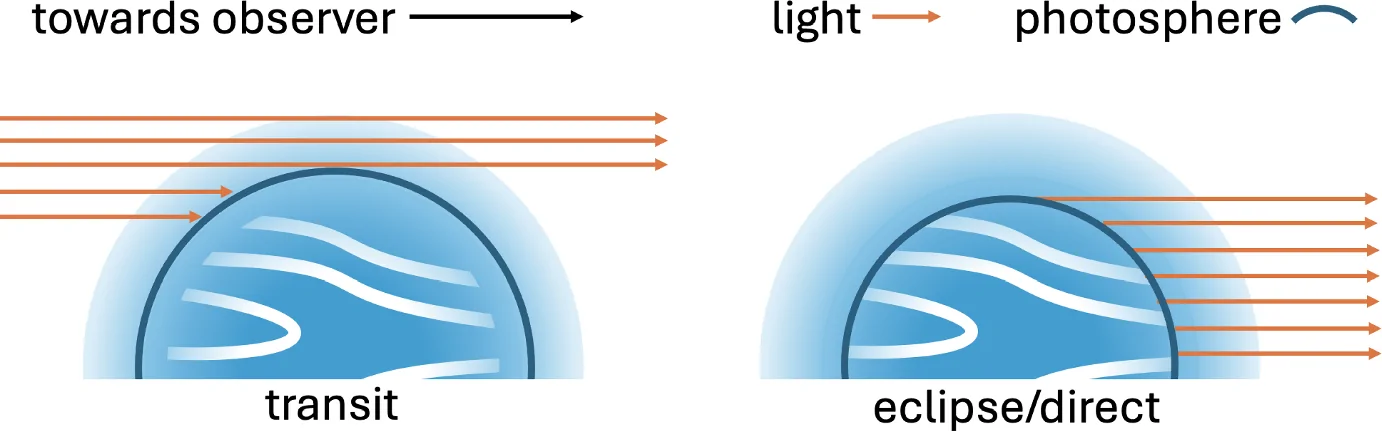
This diagram shows the difference in atmospheric path length between a transit observation (left) and an observation taken during eclipse or via direct imaging (right). The much longer path length in transit results in the photosphere (the region where the atmosphere becomes optically thick) being much higher up, because optical thickness is reached at lower pressures

The nature of the path *s* through the atmosphere will depend on the type of observation, each of which has a distinct geometry. In transit, for example, the path traversed by the light enters at the top of the atmosphere at the dayside portion of the terminator (the region between the day and night hemispheres), and exits on the opposite side of the terminator. This results in a relatively long path length through the atmosphere compared with a secondary eclipse or direct imaging observation, both of which measure the light emerging from the deep atmosphere of the planet at a range of zenith angles, averaging 45$^{\circ }$ (Fig. 2). The photosphere for the transit therefore sits at a higher altitude than for eclipse. For eclipse or direct imaging observations in reflected light, the path length is doubled to account for the path of the incoming starlight.

There are many common approximations that can be made to solve this equation, including assuming that the atmosphere is plane parallel (atmospheric state changes in only one direction), and assuming either that there is no scattering or all scattering is isotropic. Some of these approximations result in large improvements in computational efficiency, which have to be tensioned against potential loss of accuracy in the model, and some of these will be discussed further within this section. The wide range of possible approximations means that no two retrieval codes are likely to represent radiative transfer in exactly the same way, which can present challenges for benchmarking and also lead to varying efficiency of the calculation.

### Altitude grid

The temperature-pressure-altitude profile underpins the model atmosphere. Whilst recent developments have seen this extended to two or even three dimensions (see Sect. 3.8.2), here we consider the vertical dimension only, and first we discuss the altitude-pressure grid.

The altitude-pressure relation is set assuming hydrostatic equilibrium:

5 $$\begin{aligned} P = P_0 e^{-(z-z_0)/H} \,, \end{aligned}$$

where *P* is pressure and altitude is *z*, with the subscript 0 indicating values at the bottom of the atmosphere. The rate at which pressure decreases as a function of altitude is set by the atmospheric scale height *H*:

6 $$\begin{aligned} H = \frac{kT}{{\mu }g} \,, \end{aligned}$$

where *k* is the Boltzmann constant, *T* is atmospheric temperature, μ is the mean molecular weight of the atmosphere and *g* is the acceleration due to gravity. The scale height has an especially significant effect on retrievals for transit spectroscopy observations, because this quantity determines the amplitude of the spectral features. The scale height is not constant with altitude, since it depends on the temperature, but also because the gravitational acceleration decreases with increasing distance from the planet’s centre.

In transmission geometry, there is a degeneracy between temperature and gravity inherent in the scale height calculation above. The so-called ‘normalization degeneracy’ (Benneke and Seager 2012; Griffith 2014; Heng and Kitzmann 2017) arises from the fact that, whilst the mass of the planet can be independently determined from radial velocity measurements, the measured white light radius of the planet obtained in transit is for an unknown pressure level in the atmosphere, that is highly dependent on the atmospheric characteristics. In the context of atmospheric retrievals, therefore, either the planetary radius at some fixed pressure or, alternatively, the pressure at the white light measured radius must be free parameters. Whilst these approaches both have the effect of allowing the model spectrum to effectively be re-normalized to the baseline of the data, they are not exactly equivalent, which becomes clear in the example below.

Recently, analysis of the Transiting Exoplanet Community JWST Early Release Science observations of WASP-39b highlighted that, for spectra of the quality obtained by JWST, the normalization degeneracy is even more insidious. Retrieving only either the pressure or the radius neglects the fact that what determines the planet’s gravitational acceleration is the radius that encloses the measured mass. Varying the reference radius will alter the gravitational acceleration at a given altitude in a subtly different way to varying the reference pressure. Therefore, despite the fact that the parameters have a high degree of degeneracy, it may be necessary to allow both quantities to be retrieved independently in order to accurately determine the atmospheric scale height (Welbanks 2025).

The scale height is also heavily influenced by the molecular weight of the atmosphere. The molecular weight depends on the dominant chemistry of the atmosphere and thus is affected by the atmospheric metallicity, with higher metallicity atmospheres having a higher molecular weight. This quantity can also vary as a function of altitude (discussed further in Sect. 3.4). Higher molecular weight atmospheres have smaller scale heights, making them more challenging to characterize in transmission.

Finally, the scale height is affected by the temperature. Hotter atmospheres have larger scale heights. Very hot atmospheres can be substantially inflated, and this also exacerbates the change in scale height with altitude as the gravitational acceleration is further decreased in the outer layers. This dependence on temperature can also mean that the scale height varies from one side of the planet to the other; ultrahot Jupiters, with large day-night temperature gradients, may have dayside atmospheres that are substantially inflated over that of the nightside. This can lead to biases when interpreting transit spectra under the assumption that the atmosphere is homogeneous (Caldas et al. 2019; Pluriel et al. 2020). The pressure-temperature profile is therefore key to determining the overall structure of the atmosphere.

### Temperature-pressure structure

For early transit spectra obtained by Hubble, the altitude range probed by the observation was relatively small, and because of this an isothermal temperature profile was generally considered to be adequate. However, either for observations covering a wider wavelength range or at higher spectral resolution, the observation will probe a wider altitude range and therefore the isothermal approximation is no longer valid. The temperature must be allowed to vary as a function of altitude. Two main parameterization approaches are generally used, these being the Guillot profile (5 independent parameters, which may be reduced to 3 for simplified versions; Guillot 2010) or the 6-parameter profile pioneered by Madhusudhan and Seager (2009) (MS09). The Guillot profile is derived from the Eddington approximation to the radiative transfer equation, and the temperature profile is constructed based on the balance of incoming starlight in the optical and thermal emission from the planet in the infrared. Tunable parameters are the opacities in two optical streams (one for the three-parameter version); the opacity in the infrared; the ratio of the flux between the two optical streams (not included in the three-parameter version); and a measure of the atmospheric recirculation efficiency. This lends the Guillot profile a physical basis, but it is also more restrictive about possible T-P profile shapes than MS09, which is a purely parametric curve-fitting approach. For a more detailed description of how each of these is constructed and a discussion of their merits in a retrieval scheme, see Barstow and Heng (2020). Whilst both of these approaches have the advantage of only a small number of free parameters, both are somewhat restrictive about the shape of the T-P profile, which if not representative of the true temperature pressure profile may introduce biases into the retrieval.

**Fig. 3:**
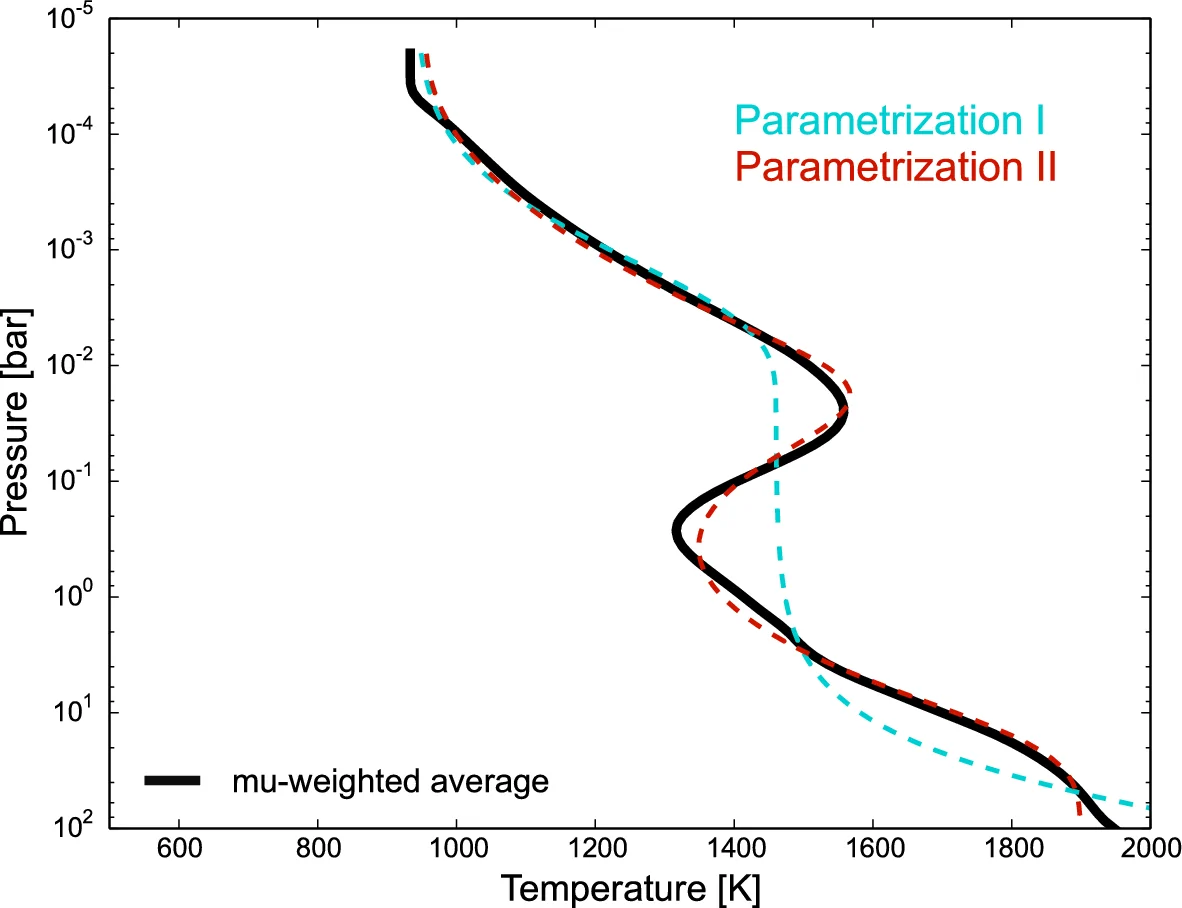
This figure shows a 3D weighted average temperature profile generated using a 3D radiative hydrodynamic model (black), compared with parameterized profiles fit to synthetic data using the Guillot (turquoise) and MS09 (red) parameterizations. In this case, the MS09 profile is able to capture the curvature in the middle part of the atmosphere much more effectively due to its increased flexibility in this region. Figure uses data taken from Blecic et al. (2017)

Blecic et al. (2017) investigate the impact of retrieving a single, representative temperature profile when the structure is in reality varying spatially over the atmospheric region probed by the observation. They also test the ability of both the Guillot and MS09 models to reproduce both the arithmetic average and emission angle-weighted average profiles. They find that, despite the weighted average being probably a more realistic representation of 3D structure, the arithmetic average is more closely matched by the retrieved profiles with either parameterization. They also find that the Guillot profile can struggle to represent curvature in the T-P profile in the middle part of the atmosphere, whereas the MS09 profile does not represent curvature in the lower part of the atmosphere. We present an example from Blecic et al. (2017) showing the performance of the Guillot and MS09 profiles when fitting an averaged profile with substantial curvature in the middle atmosphere (Fig. 3 ).

Solar System retrieval codes (e.g. Irwin et al. 2008) generally make use of more informative priors and retrieve temperature in each model layer, with a smoothing length to ensure no unphysical oscillations in the profile or model overfitting. This approach may become more feasible for exoplanet secondary eclipse spectra, which probe a broader altitude range than transmission, especially with the advent of new JWST data. Whilst this provides significantly greater flexibility, it also represents a large increase in the number of parameters in the model (typically, Solar System codes use optimal estimation Rodgers 2000, for which large numbers of parameters are not prohibitive.)

Therefore, continuous temperature profile retrievals are intractable for nested sampling. Optimal estimation retrievals, for which continuous temperature parameterization is viable, are generally not considered to be as useful as fully Bayesian methods for low resolution exoplanet spectra, due to restrictive priors and an inability to extract statistical limits on a fully marginalized solution (e.g. Line et al. 2013; Pinhas et al. 2019). The trade off between model complexity and statistical completeness means that continuous temperature retrievals for exoplanets are not currently tractable.

Recovering a good representative atmospheric structure, even in the absence of any spatial variation, therefore requires as a minimum four independent parameters (for simplified Guillot, plus a retrieval of either the radius or a reference pressure), but is more likely to need seven or eight, before we even get to retrieving gas abundances or cloud properties. This highlights the challenges of adequately describing an atmosphere in a retrieval model within the confines of samplers such as MultiNest, which will be expanded upon in Sect. 5.1.

### Chemistry

The chemical make up of the atmosphere must also be specified in the retrieval model. Again, here we will consider the vertical dimension only and discuss spatial variation in Sect. 3.8.2.

**Fig. 4:**
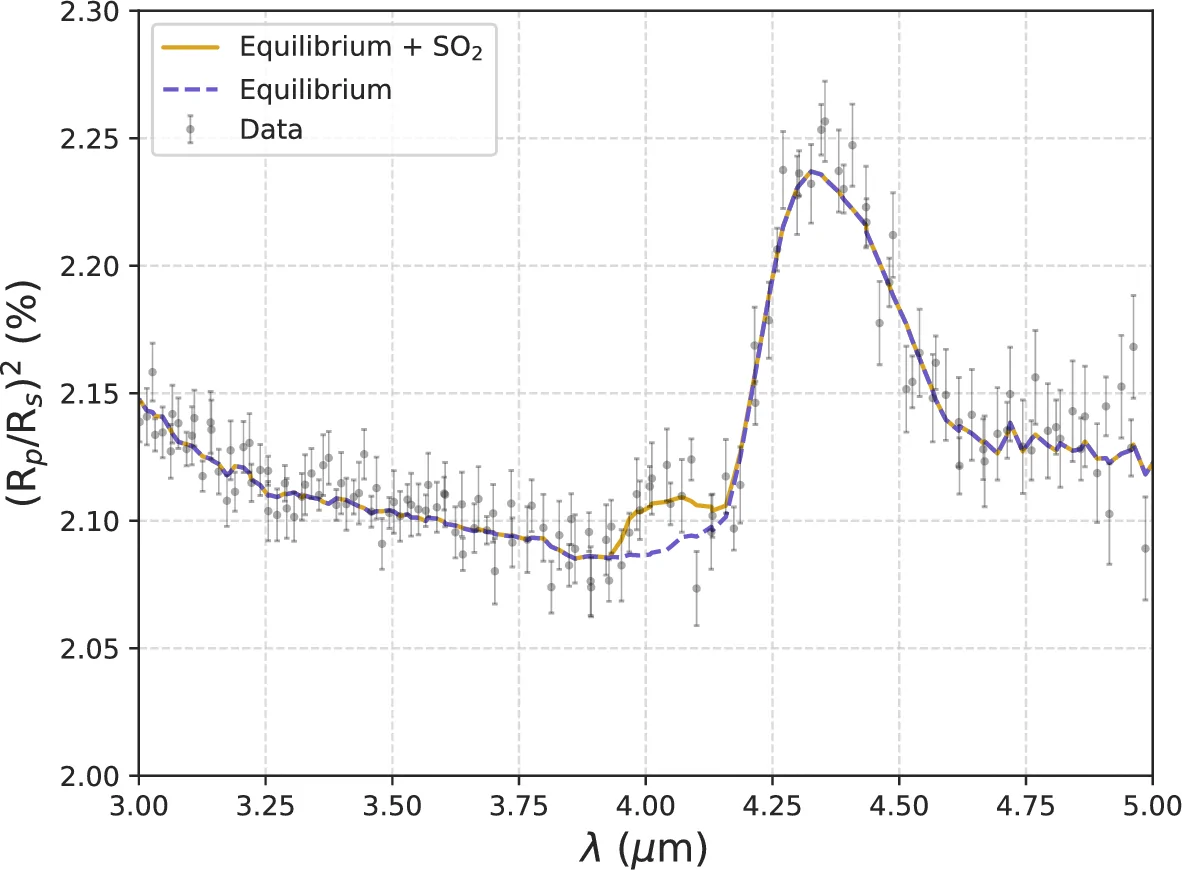
Fits with the NEMESISPY (Irwin et al. 2008; Yang et al. 2024) retrieval code coupled with FASTChem (Stock et al. 2018) to the WASP-39b ERS transmission spectrum (Carter et al. 2024). A model where the SO$_2$ abundance is allowed to vary as an additional free parameter (gold, solid) fits the data at around 4 μm much better than the pure equilibrium model (purple, dashed). There is clearly missing opacity in the equilibrium model. Figure created by Agnibha Banerjee and used with permission

The main choice when incorporating atmospheric chemistry into a retrieval is whether, or not, to impose the outcome of any self-consistent chemical network on the model atmosphere. Both approaches have advantages. Adopting a free-chemistry approach does not impose as many prior assumptions, so allows us to discover chemical combinations that we might not have expected. A good example of this is the presence of unexpectedly high amounts of SO$_2$ in the atmosphere of the JWST Early Release Science target WASP-39b (Alderson et al. 2023; Rustamkulov et al. 2023). This was not predicted by the thermochemically consistent models that were initially applied to interpretation of the dataset, but instead prompted further modelling work that uncovered a photochemical mechanism for the presence of SO$_2$ (Tsai et al. 2023). Whilst in this case the SO$_2$ feature was strong enough to identify by eye (Fig. 4), and could not be reproduced by any combination of other gases in the model, there may be scenarios where the use of a chemically consistent model would have masked a detection that would have resulted in a significant advance in understanding.

On the other hand, the inclusion of chemical networks comes with the advantage of significantly fewer free parameters being required to describe an atmosphere with even a large number of different species. Generally, for example, retrieved parameters are just the overall atmospheric metallicity, and certain specific atomic ratios such as C/O ratio. (e.g. PLATON, Zhang et al. 2019). Self-consistent chemistry also removes the necessity of assuming that gases are well-mixed in altitude, or follow a vertical profile of a particular shape, because the vertical profiles are calculated consistently. Several standalone chemical networks have been successfully coupled to retrieval codes, including FASTChem (Stock et al. 2018; Kitzmann and Stock 2018), GGChem (Woitke and Helling 2021) and ACE (Agúndez et al. 2012, 2020).

However, tying a specific chemical network to a retrieval also comes with potential complications. The authors of Al-Refaie et al. (2024) compute a synthetic spectrum using a full disequilibrium chemistry network for the underlying model atmosphere. They then perform retrievals using both the same network, and a reduced version of it. The reduced network can reproduce most of the spectrum well, but the retrieval produces erroneous values, including a metallicity of 6x solar where the input was 1x solar, whilst using the full network recovered the input parameters correctly. This analysis demonstrates that using a *suitable* chemical network can be a powerful retrieval tool, but using an *unsuitable* network can force the retrieval into a solution that is incorrect, whilst still providing an apparently good match to the measured spectrum.

As we learn more about the different processes at work in shaping exoplanet atmospheres, disequilibrium chemistry models will continue to increase in complexity. This means they are more likely to capture all necessary physics to describe a real atmosphere, but will also mean that calculation times are longer. In a retrieval scenario, the resultant model atmosphere has to be evaluated at every step. The FRECKLL model presented by Al-Refaie et al. (2024) probably contains the most complex chemical model in a retrieval so far; the authors report that the evaluation takes 5 min, and the retrieval requires a total of 40,000 samples to converge. Assuming that the evaluation of the chemical network dominates the length of a single model iteration, this corresponds to a total CPU time of 12 million seconds, or 138 days. Whilst the time pressure can be alleviated by running on large multi-core machines (the authors use 180 cores), this represents a large investment in computing time for a single retrieval.

Chemical (dis)equilibrium models also encode vertical variation in atmospheric composition. For example, we do not expect photochemically-produced SO$_2$ on WASP-39b and other similar planets to be well-mixed throughout the atmosphere, but rather confined to a relatively small pressure range where reaction rates and mixing favour SO$_2$ production. Where we expect condensational cloud to form at a particular altitude in the atmosphere, for example silicate cloud as detected on VHS 1256–1257b (Miles et al. 2023), we would expect the corresponding gas phase species (in this case, SiO, Mg and/or Fe) to be depleted.

An extreme example of this occurs for ultra hot Jupiters, where the temperature on the dayside is elevated above the thermal dissociation threshold for molecular hydrogen. In these examples, above a certain altitude the atmosphere is no longer dominated by molecular hydrogen but by atomic hydrogen (see e.g., Gapp et al. 2025). Atomic hydrogen has different continuum features, but perhaps more importantly has a molecular weight only half that of molecular hydrogen. Where thermal dissociation of hydrogen occurs, the atmospheric scale height can undergo drastic change (see Sect. 3.2).

These effects can also be represented in free retrievals by parameterizing the vertical profile of a gas in a way appropriate to the mechanism of production/destruction. For example, in Gapp et al. (2025) the dissociation of molecular hydrogen and of water vapour are both parameterized in the retrieval according to the formalism outlined by Parmentier et al. (2018), where the gas abundance starts to decline at a particular pressure and then continues to fall off with decreasing pressure according to a power law. Depletion due to cloud condensation could also be parameterized similarly, by linking e.g. the retrieved pressure of the cloud base to the pressure below which the molecule is removed from the gas phase. All of these parameterizations add to the number of variables within the model and increase the overall complexity, leading to longer retrieval times; however, some are already necessary, and others are likely to become so as quality and coverage of data increases.

### Opacities

The use of appropriate molecular opacities as an input for a retrieval model is critical for accurate retrievals of exoplanet atmospheres. This need comes with its own significant computational burden. Exoplanet atmospheres are generally substantially hotter than conditions that can easily and safely be replicated in the laboratory, so predictions of absorption line positions and strength rely on quantum mechanical simulations to generate rovibrational energies, using software such as TROVE (Tennyson and Yurchenko 2017).

The most accurate option for incorporating opacity data into a model is a full line-by-line calculation, for which the broadening and absorption due to each spectral line is calculated individually, with the overlap between lines of different species taken into account on the fly. However, this is an extremely computationally intensive process, so much so that line-by-line calculations are generally not performed for retrievals, since they are intractably slow. Instead, two different approximations to this calculation are in common use — cross sections and k-tables.

Correlated-k tables work by splitting the spectrum into wavelength intervals of a specified resolution, and then defining a function for the cumulative absorption as a function of the fraction of the wavelength interval. This produces a smooth function that can be sampled and stored in look up tables, called k-tables, which vastly simplifies the integral of optical depth over each wavelength interval. The correlated’ part of the name arises from the implicit assumption that the strongest lines in each layer of the atmosphere will be well correlated with the strongest features in adjacent layers.

Whilst the correlated-k technique allows for relatively rapid computation, there is a cost in accuracy that can be of order several percent. There are also challenges when combining k-tables for different gases that have overlapping strong features. Many codes adopt the random overlap method, which assumes that the strongest features from different gases are uncorrelated; other treatments are available, such as the equivalent extinction method adopted by Amundsen et al. (2017), which is less accurate than random overlap but can be computed more rapidly and is therefore more useful for computationally intensive applications such as global circulation models.

**Fig. 5:**
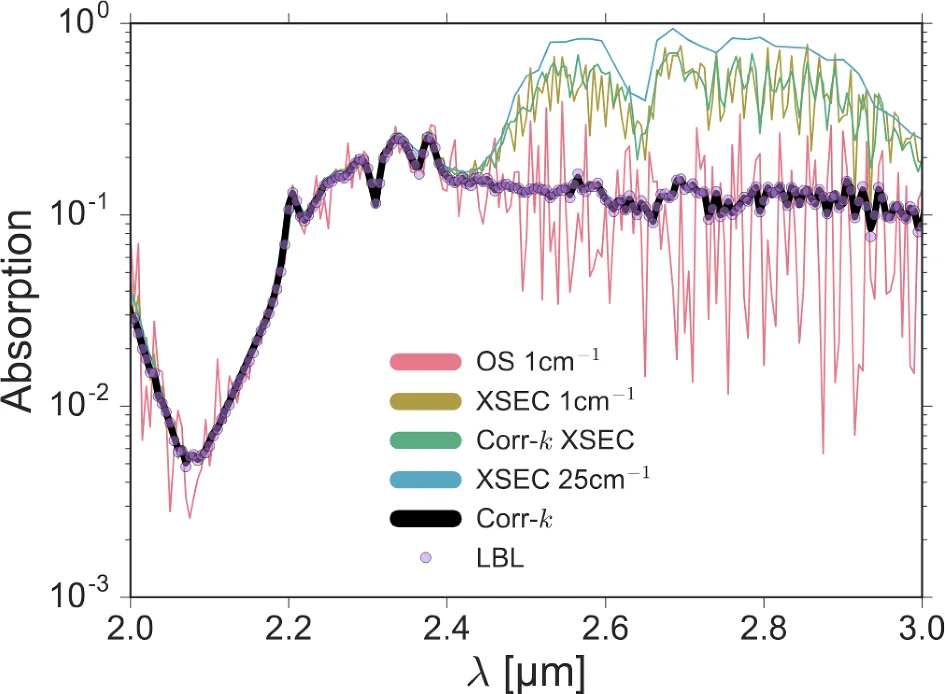
This figure is taken from Garland and Irwin (2019) and shows the performance of different opacity sampling methods compared with a full line-by-line calculation, for a layer consisting of 50% CH$_{4}$, 50% H$_{2}$O at 1000 K and path amount of $1 \times 10^{20}$ molecule cm$^{-2}$. Pink is opacity-sampling at 1 cm$^{-1}$ resolution using the 0.01 cm$^{-1}$ resolution ExoMol cross-sections. Gold and blue are the cross-section absorption spectra at 1 cm$^{-1}$ and 25 cm$^{-1}$ resolution respectively, the black line is the correlated-k method with a resolution of 0.002 μm and the green line is the cross-section derived from the Garland and Irwin (2019) correlated-k method. The correlated-k method clearly provides by far the best match to the line-by-line calculation

An alternative approximation is to generate absorption cross section tables. This method also produces a pre-tabulated grid over temperature and pressure space, but does not perform the coordinate transform included in the correlated-k method. Typically, the underlying spectral resolution of the cross sections must be high to maintain a reasonable level of accuracy against a line-by-line calculation, whereas correlated-k tables computed much closer to the resolution of the data perform well against the line-by-line solution (Garland and Irwin 2019; Leconte 2021, Fig. 5). This difficulty specific to cross-section resolution is highlighted by Welbanks et al. (2026) in their analysis of the combined WASP-39 b JWST dataset binned to R∼100; tests conducted with the PyRatBay (Cubillos and Blecic 2021) and CHIMERA (Line et al. 2013) retrieval codes, revealed that retrieved gas abundances were biased for cross-sections computed as a resolution lower than R∼50k (PyRatBay) and R∼5k (CHIMERA). Cross-sections calculated at high resolution may be subsequently down-sampled using the opacity sampling method (red line in Fig. 5), which is a less biased approach than using cross-sections calculated at low resolution but does introduce more scatter than for a correlated-k spectrum at the equivalent resolution.

Regardless of the approximation adopted, the step of calculating the correlated-k and cross-section tables also comes with computational challenges. Whilst quantum mechanical simulations can predict the location and strength of transitions, in a real atmosphere each line is subject to broadening. The shape of the broadened line is generally described by a Voigt profile — a convolution of a Lorentz profile, representing the pressure-broadening, and a Gaussian, representing the Doppler broadening. In the lower part of an atmosphere, at higher pressures, the profile is close to Lorentzian and in the upper atmosphere it is dominated by Doppler broadening and is more Gaussian in shape. The width of the Lorentzian function depends on the bulk atmospheric composition; line broadening in (Earth) air is considerably different to the broadening that is expected in a gas giant atmosphere dominated by hydrogen and helium. Room-temperature broadening coefficients in different bulk gases can be measured in the lab, but they are temperature-dependent and extending them to higher temperatures needs to be parameterised in some way. These coefficients also depend on the rotational and vibrational quantum numbers, but the latter is usually ignored for simplicity since it has a smaller effect than the rotation, and only limited data are available on the former; this introduces a further source of potential inaccuracy in modelling of spectal absorption features. Databases such as HITRAN now provide semi-empirical broadening coefficients for a range of background gases (e.g., Tan et al. 2022).

The wings of a theoretical Voigt profile extend an infinite distance away from the line centre, so it is usually calculated out to a specified distance in wavenumber space away from the line centre. A typical cut off distance is 25 cm$^{-1}$, since for the majority of species the absorption of the broadened line beyond this is very close to zero. However, there are notable exceptions to this, including the atomic absorption features due to Na and K in the visible part of the spectrum. The wings of these lines can be very asymmetric and in the pressure broadened region they can extend much further from the line centre in terms of significant absorption compared with molecular species (e.g., Welbanks et al. 2019).

For some species, such as CH$_4$, high temperature databases contain a vast number of individually weak lines, that nonetheless can contribute substantial opacity when combined. Calculating cross-section and correlated-k lookup tables incorporating each of these lines individually can be very intractable. One solution is to subsume all weak lines below a certain threshold into a pseudo-continuum, a process dubbed ‘superlines’ (Yurchenko et al. 2017). In this way, the cumulative opacity from weak lines can be incorporated without unnecessary computational burden.

All of the factors above are considerations when computing the cross-section or correlated-k databases for use in retrievals. Several different compromises between accuracy and computational viability must be made at different steps.

### Clouds

Clouds and hazes represent possibly the most complex aspect of atmospheric modelling for retrievals. They have proved to be virtually ubiquitous for worlds with substantial atmospheres, as their presence cannot be ruled out even for ultrahot Jupiters such as WASP-76b, where there is evidence for condensates forming on the nightside (Ehrenreich et al. 2020). The exoplanet WASP-96b, originally thought to be cloud-free, has also shown evidence for the presence of clouds in the transmission spectrum obtained by JWST (Taylor et al. 2023).

Aerosols are a particular challenge for retrievals due to the number of factors that influence their interaction with light. Their imprint on a spectrum varies with their location (vertically and spatially), the size/aspect ratio of the particles or droplets, what those particles/droplets are made of, and the amount of particles/droplets that are present. Prior knowledge about aerosol composition and size distribution is extremely limited for exoplanets. Whilst it is possible to simulate these using cloud microphysics models (e.g. simulations with CARMA, Gao et al. 2020) it is worth noting that attempts to *a priori* model the clouds on Jupiter and Saturn do not produce a solution that is consistent with observations. Clouds on Jupiter and Saturn are predicted to be made of ammonia and ammonium hydrosulphide, but in practice ammonia clouds are not spectrally identified across most of Jupiter (Atreya and Wong 2005), and on Saturn the tropospheric cloud deck doesn’t sit at the predicted pressure level for either species (Barstow et al. 2016). Therefore, predicting the location, size distribution and composition of aerosols on exoplanets, for which our understanding of cloud formation processes is much more lacking than in the Solar System, cannot be assumed to be successful.

The multiplicity of cloud types for a single planet is also a complicating factor. As well as the giant planets, which have different cloud species condensing out at different altitudes, Venus is an example of a remarkably complex cloud structure involving only a single species. Venus has two distinct decks of sulphuric acid cloud, made up of four separate particle size populations (e.g., Crisp 1986; Pollack et al. 1993; Grinspoon et al. 1993; Barstow et al. 2012). The vertical positions and relative number densities of these populations have an observable effect on infrared spectra.

By contrast, aerosol models for exoplanet retrievals are, so far, crudely simplified. Typical parameterizations specify a cloud top pressure (and possibly cloud base pressure), and optical depth with wavelength dependence that follows some power law (e.g., Pinhas et al. 2019; MacDonald and Madhusudhan 2017; Barstow et al. 2017). Others make an attempt to recover some more meaningful information about the optical properties of the particles that may be related back to their size or composition, for example the scheme proposed by Kitzmann and Heng (2018) where they find an analytical model format that produces reasonable fits to calculated extinction properties for a range of cloud species. In a similar vein, Taylor et al. (2021) fit a simple function for the single-scattering albedo of dayside cloud particles, rather than assuming a power law type relationship.

More recently, with the detection of distinct silicate cloud features in JWST spectra of some planets (e.g. Grant et al. 2023; Dyrek et al. 2024), retrievals have evolved to model specific cloud species using look up tables (e.g. Mullens et al. 2024). This has the advantage of being able to reproduce absorption features without substantially increasing the number of fitted parameters, but it limits an individual retrieval run to a specific cloud species. Including instances where clouds may be a mixture of components at the grain level (e.g. Helling et al. 2008; Kiefer et al. 2024) is also not possible with this approach.

Irwin et al. (2015) apply a more flexible approach to retrieving ground-based spectra of Uranus. They use the Kramers-Kronig relation to retrieve the complex refractive index using a real-time Mie theory calculation. The retrieved refractive index can then be compared with the known refractive indices of different potential cloud species to determine the composition. This is appropriate to Optimal Estimation retrievals within the solar system context, but would be prohibitively slow if applied to exoplanets in this form, due to the need to calculate Mie scattering at every step.

**Fig. 6:**
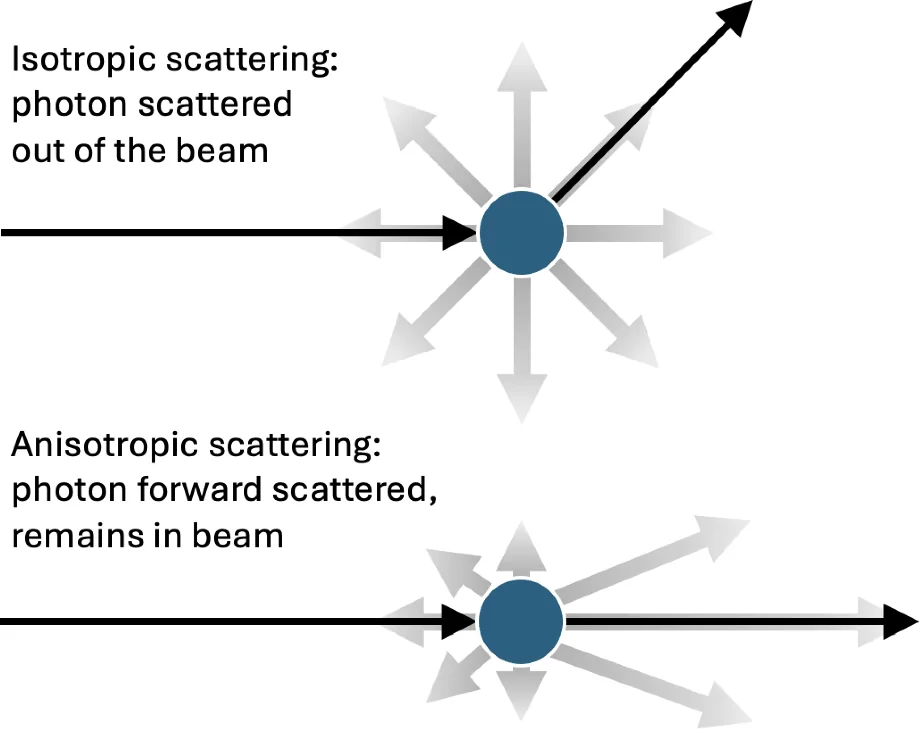
The scattering properties of the cloud dictate the utility of the extinction-only approximation for a particular case. For isotropic scatters (top) the approximation holds reasonably well, but for highly anisotropic scattering (bottom), especially dominated by forward scattering, the extinction-only approximation substantially underestimates transmission through the atmosphere

Another major simplification that is commonly made within exoplanet retrievals is to use an extinction-only approximation for clouds; this assumes that a photon encountering a cloud or haze particle is always scattered out of the beam, and does not require a full multiple-scattering calculation to be performed. This is often not the case in practice, however, since strongly peaked scattering can result in photons being preferentially scattered at angles of either 0$^{\circ }$ or 180$^{\circ }$ (Fig. 6). Photons that experience a combination of forward- and back-scattering events in this way tend to remain in the beam and do eventually emerge from the atmosphere in the line of sight, albeit having experienced a longer atmospheric path. de Kok and Stam (2012) test this assumption, and note that the atmospheric transmission is underestimated at high optical depths when multiple scattering is ignored. However, correctly simulating multiple scattering in transit geometry is particularly computationally intensive, requiring Monte Carlo radiative transfer modelling tracking individual photon paths, and whilst it is more feasible for emission or reflection geometry via a matrix operator algorithm (e.g., Plass et al. 1973) it still substantially increases the computation time for a single model iteration. This represents a barrier to more accurate cloud modelling, especially where forward scattering dominates.

### Model binning

In order to compare each model instance with the observed data and calculate the likelihood, the model must be binned to the same wavelength grid as the data. There are several modelling choices to be made in the way that the binning is carried out, and this can also introduce further bias into the retrieval.

Deming and Sheppard (2017) introduce the problem of resolution linked bias in the context of transit spectroscopy data. In their Eq. 4, they express the measured transit signal $S_{\lambda }$ as follows:

7 $$\begin{aligned} S_{\lambda } = 1-\frac{I_{\lambda }\otimes (F_{\lambda }(1-A_{\lambda }))}{I_{\lambda }\otimes F_{\lambda }} \,, \end{aligned}$$

where $F_{\lambda }$ is the stellar flux at wavelength λ, $A_{\lambda }$ is the flux absorbed by the planet, and I$_{\lambda }$ is the instrumental response function. This equation is not equivalent to the standard approach adopted (Deming and Sheppard 2017, their Eq. 5) which simply represents the stellar flux using a low-resolution (effectively pre-binned) stellar spectrum, cancels the stellar flux terms in the above equation and convolves the absorption $A_{\lambda }$ with the instrument response after the fact. If there are many strong lines in the stellar spectrum, as is the case for M stars, this process can introduce substantial bias in the calculated wavelength-dependent transit depth.

Similar considerations apply in calculating the atmospheric model. For relatively high resolution datasets, such as those obtained using JWST, it is common practice to simply bin data using a boxcar of the appropriate width. Some codes pre-average k tables and actually perform the model calculation at the resolution of the data, but this approach is not feasible for cross-sections as explained in Sect. 3.5 above. These two approaches may also have subtle differences in the treatment of opacities outside of the k-table/cross-section framework, e.g. collision-induced absorption and cloud opacities; however, both of these typically have smoother and more slowly varying functions than molecular and atomic line opacities, so bias should be minimal.

For datasets obtained using older space-based instruments such as Spitzer, or for some low-resolution ground-based telescopes, assuming a boxcar is insufficient. For example, channels in the Spitzer IRAC instrument have very distinctive instrument response functions that are not a boxcar or a Gaussian. In this case, it is necessary to explicitly convolve the model in the Spitzer bandpasses with the correct instrument response function, since not doing so can incorrectly weight the underlying high resolution spectrum and sometimes result in substantially different results.

The problem described above of resolution linked bias in generating a transit spectrum from observations also emerges when we consider models of eclipses and phase curves. Here, the radiative transfer calculation we perform generates the synthetic spectrum for the planet, but what is measured is the ratio of this over the spectrum of the star. Usual practice is to divide the calculated planetary spectrum by a stellar spectrum obtained from a library, e.g. PHOENIX (Husser et al. 2013); however, if the stellar spectrum and the planetary spectrum are pre-convolved/binned this will be subject to the same bias as the calculated transit depth in the above example. The most accurate approach would therefore be to calculate the planetary spectrum at the same high resolution as the stellar spectrum model, take the ratio and then only afterwards bin/convolve to the instrument resolution; however, for codes that use k table averaging to improve efficiency of the radiative transfer, this would slow down the calculation substantially. Once again, we find that there is a trade space to explore between accuracy and efficiency.

Finally, Davey et al. (2025a) examine the impact of binning down both data and models on retrievals in the presence of clouds at various altitudes. They find that for cloud at very hight altitudes/low pressures, binning down to a lower than native resolution limits the accuracy to which the atmospheric state can be recovered, because the only molecular features visible are narrow features that can still be seen above the cloud opacity floor. Binning should therefore always be approached with care.

### Other considerations

#### Stellar heterogeneities

**Fig. 7:**
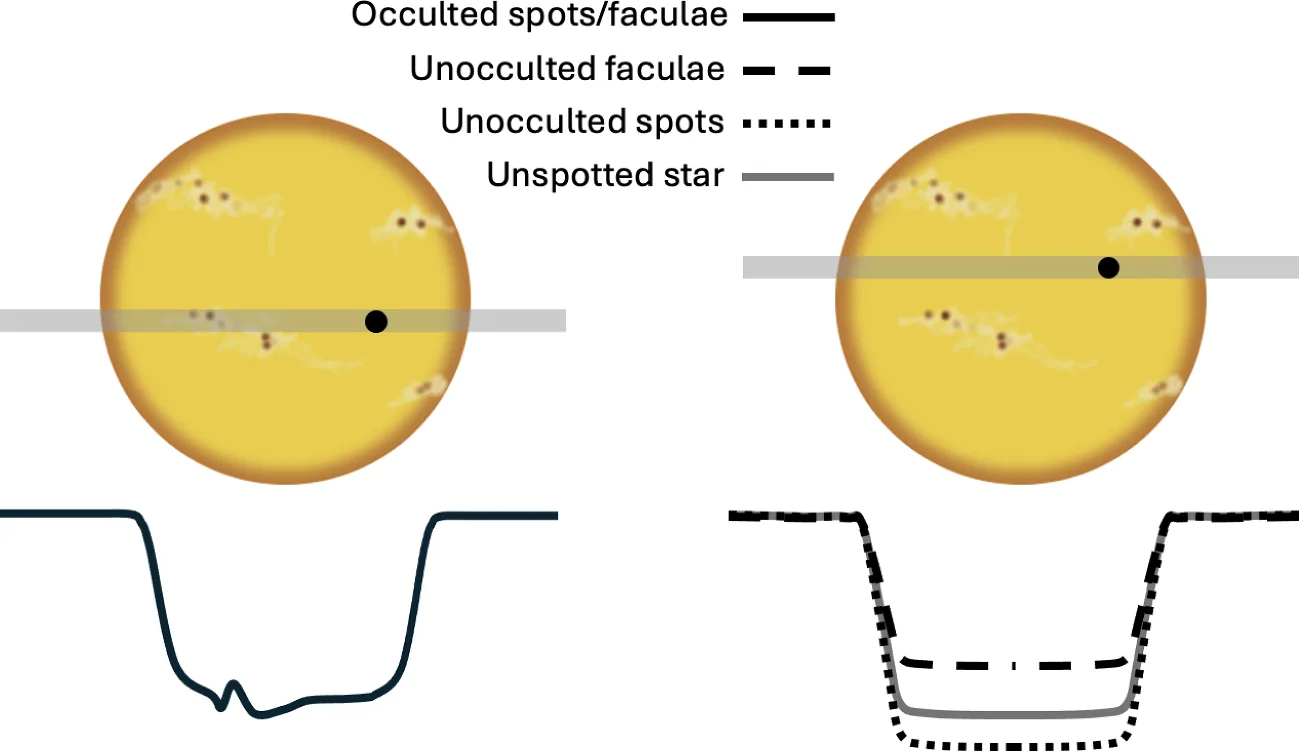
The presence of starspots and faculae on the surface of the star can result in transits and resultant spectra being contaminated by the ‘Transit Light Source Effect’. This occurs when the planet either occults faculae and spots, introducing additional features into the lightcurve (left) or occults a pristine area of the stellar disc, altering the overall transit depth due to the occulted region being unrepresentative of the disc average (right). Unocculted starspots result in the transit appearing deeper, whereas if the signal is dominated by unocculted faculae the transit will appear shallower. The effect is chromatic, with the spot/faculae/photosphere contrast increasing towards shorter wavelengths

So far, we have focused on planetary parameters in our analysis of retrieval challenges. For many targets, the star itself can present a substantial difficulty to accurate astrophysical characterization. The Transit Light Source Effect (Rackham et al. 2018) refers to spectral imprints arising from heterogeneities on the surface of the star, either spots, faculae or a mixture of both. When a planet transits, if the transit chord spot/faculae for consistency coverage is not representative of the overall stellar disc, then this results in the spectral spot/faculae contrast with the pristine photosphere being imprinted onto the transit spectrum (Fig. 7). The degree to which this impacts the spectrum depends on the spot/faculae coverage and their temperatures relative to the photosphere.

Whilst the relative level of spot coverage at a given time can be determined from ground-based monitoring of the star, e.g. as used by Pont et al. (2013), this does not constrain the baseline filling factor. Therefore, more recent works have attempted a simultaneous correction for spot/faculae parameters within the retrieval itself (e.g. Fournier-Tondreau et al. 2024). These approaches typically use standard stellar atmosphere model grids such as PHOENIX (Husser et al. 2013), and make the implicit assumption that the spot or facula can be represented by a stellar atmosphere model of the appropriate temperature. Spot retrieval parameters for the simplest version include the star’s photospheric temperature (usually with an informative Gaussian prior, since this is known to first order); the spot/facula temperature; and the filling factor. More sophisticated models may specify both spots and faculae, thus having a temperature and a filling factor for each and increasing the number of parameters to five. Fournier-Tondreau et al. (2024) also investigate whether spot spectra are better represented by models with a different log(g) value as well as a different temperature, further increasing the number of parameters. An alternative approach used by Thompson et al. (2024) is to fit for spot temperature, radius and stellar latitude assuming a single spot; whilst this approach also uses PHOENIX models, it accounts for the projected spot area on the surface and also the effects of stellar limb darkening on the spectra.

A moderate increase in the number of retrieval parameters is therefore a necessary evil if spot corrections are required, which they typically are for active late-type K stars and M dwarfs. Perhaps a more significant issue is that standard stellar spectra models such as PHOENIX are not necessarily representative of spot and faculae spectra. Smitha et al. (2025) simulate spots with 3D magnetohydrodynamic simulations, and then generate the expected spot spectra; Norris et al. (2023) perform the same exercise for faculae. They demonstrate that standard stellar atmosphere models for the same spot/facula temperature have different spectral characteristics to these more physically motivated models, with spot contrast deviations between these model types exceeding 100 ppm at some wavelengths (Smitha et al. 2025). Magnetohydrodynamic simulations are too slow to be performed on the fly as part of a retrieval; other methods that better represent spot spectra within a retrieval need to be explored and this is an area of ongoing research within several teams.

The requirement to correct for spots also enforces different observational approaches. Whilst spot effects are apparent in the near-infrared and can dramatically affect spectra obtained with instruments such as NIRISS on JWST (e.g. Lim et al. 2023), diagnostic spectral features that can in particular help to discriminate between the effects of spots and faculae are present at the shorter wavelengths, currently only accessible in space to Hubble. Since spot coverage changes as the star rotates, this highlights a potential need for simultaneous Hubble and JWST observations, which is logistically demanding. Stellar heterogeneity correction represents a challenge beyond the realm of spectral retrieval and is a limiting factor on the accuracy with which atmospheric properties can be recovered from transmission spectra.

#### Multi-dimensional retrievals

In the light of recent observations from JWST (Espinoza et al. 2024) it is increasingly apparent that assuming the observed portion of the atmosphere is homogeneous is no longer a valid assumption. Whilst it has become relatively common practice to allow for inhomogeneous terminator cloud coverage when modelling transit spectra (e.g., Line and Parmentier 2016), it is becoming clear that the temperature and chemistry can also be highly variable around the terminator region. Conditions can also vary significantly along the line of sight, especially for ultrahot Jupiters where very strong day-night temperature gradients can result in large changes to the atmospheric structure between the day and night regions, which can certainly impact the spectrum (e.g., Caldas et al. 2019; Pluriel et al. 2020). Some retrieval approaches have been developed to confront this (e.g., Lacy and Burrows 2020), with several building on the combination of multiple 1D spectra (as in e.g., Line and Parmentier 2016), sometimes called the 1+1D approach, to produce a pseudo-multidimensional model (e.g., Espinoza and Jones 2021; Welbanks and Madhusudhan 2022). Recent analysis of the transmission spectrum of ultrahot Jupiter WASP-121b (Gapp et al. 2025) included a very simple two-component model, allowing the temperature profiles for the day and nightside halves of the terminator to be retrieved separately, and also allowing for differences in chemistry between the two. This resulted in the finding that water vapour is dissociated on the dayside but present on the nightside; this provides an effective spectral window in the water band that allows the nightside portion of the terminator to be probed, despite the greater extent of the more inflated dayside atmosphere. One may therefore extract information about the ‘day’ and ‘night’ regions of the terminator from a single transit spectrum, provided that chemical differences between ‘day’ and ‘night’ result in a ‘day’ spectroscopic window at wavelengths where ‘night’ absorption is present. This type of simple day-night model is not substantially slower to compute than a homogeneous atmosphere model.

More complex representations have also been developed. MacDonald and Lewis (2022) introduce a 3D radiative transfer model, TRIDENT, that encompasses both morning-evening and day-night gradients using linear algebra to compute the radiative transfer across the day-night divide and around the inhomogeneous terminator. Whilst the 1D baseline model calculation is very rapid, taking only 70 ms, the computation time increases with the number of day-night segments and azimuthal sectors required for the 3D calculation. The authors recommend a minimum of 6 segments and 4 sectors for a full 3D calculation, which results in a factor ∼25 slowdown. It is clear to see that, whilst the data increasingly demand it, incorporating 3D effects in radiative transfer models substantially increase the computational burden. Nixon and Madhusudhan (2022) introduce a similar framework. AURA3D, which computes the atmospheric temperature structure at 3 angles across the day-night terminator region, and can also account for azimuthal variation. This results in fitting for 3 T-P profiles instead of one, although the deep temperature is constrained to be homogeneous so the total number of parameters increases from 6 to 14.

Extracting 3D information from a transit observation, where each optical path passes through all the different atmospheric regions we are trying to disentangle, is therefore challenging. However, this is not our only pathway to obtaining 3D information about planetary atmospheres. Atmospheric phase curve observations contain explicit information about the zonal (longitudinal) structure of an exoplanet, and in some retrieval codes specific adaptations are now implemented to model phase curve observations. Basic phase curve retrievals simply assume a 1D homogeneous atmosphere for each phase bin (e.g., Stevenson et al. 2014; Bell et al. 2024), which neglects the fact that the atmosphere will likely show substantial variation across the disc at each phase. New retrieval techniques are now being developed to analyse the phase curve as a whole, which implicitly accounts for this variation. Representations include the TauREx 1.5D mode (Changeat and Al-Refaie 2020) which divides the planet up into dayside, nightside and terminator regions with distinct atmospheric conditions on each; the NEMESIS 2.5D mode (Irwin et al. 2020), which adopts a geometric function to govern the latitudinal variation but is able to retrieve the temperature as a function of longitude (and could in principle also retrieve gas abundances in the same way); and the NEMESISPY extension to this (Yang et al. 2023), which parameterises the dayside and nightside temperatures for a more rapid computation. In contrast with 3D modelling approaches for the terminator spectrum, these frameworks are not necessarily more computationally intensive than performing 1D retrievals, since in the 1D case individual retrievals need to be performed for each phase-resolved spectrum, whereas specially adapted phase curve retrievals fit for all phases simultaneously.

The current quality of data now means that for many planets 3D information is recoverable from transit spectra as well as phase curves, and this additional model complexity is likely to be necessary to adequately explain the data. Determining the appropriate level of complexity for a given dataset will be an important consideration to ensure computational efficiency. Testing retrieval frameworks on 3D GCM simulations as in e.g. Irwin et al. (2020); Yang et al. (2023) will be a valuable research avenue to resolve this.

### Machine learning emulators for radiative transfer

It should now be clear that different aspects of the radiative transfer and atmospheric modeling can introduce a great deal of computational complexity, and this will have the effect of slowing down the retrieval calculation. Above, we identify several specific areas that can act as bottlenecks to an efficient retrieval process. One approach that is beginning to gain traction to mitigate these issues is the use of machine learning tools as emulators for aspects of the modeling process. If these emulators are able to reliably learn and replicate a particularly computationally intensive part of the model, then this could result in a substantial speed-up in the model run time. Examples of this currently in use include Schneider et al. (2024), which uses a neural network to compute gas absorption where features overlap, and Himes et al. (2022), in which the neural network emulates the whole radiative transfer calculation.

Schneider et al. (2024) examine the challenge of correctly combining k table absorption from different gases in the context of a Global Climate Model (GCM) rather than a retrieval; however, the method could just as easily be applied in a retrieval context. The most accurate method for combining k tables with multiple overlapping lines from different species is the random overlap with rebinning and resorting (RORR) method, which stands up very well against full line by line calculations; other methods tested are shown to be prone to systematic errors. The RORR method is too computationally slow to be performed within the GCM. A machine learning method based on deep sets is shown to produce accurate output, whilst being over a factor 2 faster than RORR.

Himes et al. (2022) have taken this a step further and developed an emulator for the whole radiative transfer calculation. Their software package MARGE is trained on data generated using BART (Harrington et al. 2022), a traditional radiative transfer and retrieval package. Spectra predicted from atmospheric parameter inputs by MARGE can be compared with spectra calculated using BART, finding that the residuals between the two are on the order of a few % and are scattered around zero.

Scattering calculations form one of the biggest bottlenecks in exoplanet radiative transfer, and Dahlbüdding et al. (2024) start to address this by setting up a physics-informed neural network, or PINN, to emulate atmospheres with Rayleigh scattering included, rather than using the approximation of all scattering events resulting in scatter out of the beam. They find good agreement comparing with traditional code petitRADTRANS (Nasedkin et al. 2024a).

Overall, there are extensive machine learning applications for the radiative transfer modeling aspect of the retrieval problem. We discuss their application to the inversion aspect in Sect. 5.1.1.

## Data considerations

The data used as input to a retrieval is also an important consideration. Whilst data reduction—i.e. going from a detector image to a flux measurement—is not within the scope of this paper, converting a time series of measured fluxes to a transit lightcurve is effectively a retrieval process in its own right, and decisions made during that process can have effects on the results of atmospheric retrievals down the line. Challenges in lightcurve fitting involve stellar heterogeneities and limb darkening, and potential non-sphericity of the planet. High-resolution and direct imaging observations have their own specific challenges associated with performing retrievals, which we discuss below. For example, high-resolution ground-based data requires the removal of telluric absorption from Earth’s atmosphere before it can be used.

### Light curve fitting

The process of light curve fitting is in effect a retrieval in itself. A detailed exploration of light curve fitting techniques is beyond the scope of this review, but in brief, light curve fitting tools such as e.g. Kreidberg (2015) typically generate a number of synthetic light curves for a circular planet crossing a circular stellar disc, incorporating some parameterization for stellar limb darkening and varying the star and planet parameters for each iteration. The transit depths obtained from these light curve fits form the spectroscopic data analyzed during spectral retrieval. Therefore, the assumptions that go into the light curve fitting process can affect the result of a retrieval. Below, we highlight two examples in which a poor choice of light curve parameters can significantly impact results.

#### Non-sphericity

We discussed in Sect. 3.8.2 the challenge of modelling atmospheres where the terminator composition, and therefore opacity, is not homogeneous. An additional complication can also arise at the light curve fitting stage, as an inhomogeneous terminator can actually alter the apparent cross section of the planet from a circle to an ovoid shape. This arises from the fact that higher temperature regions of the atmosphere will have an increased scale height over cooler regions, and so warmer regions of the terminator atmosphere will be more extended than cooler regions. Additionally, higher cloud opacity on one limb than the other can also alter the shape of the planet’s opacity contours. One approach to model this is the concept of ‘transmission strings’ (Grant and Wakeford 2023), which parameterizes the angle-dependent planetary radius around the limb as a Fourier series. This technique enables an angle-dependent transmission spectrum to be recovered, leading to the opportunity for more direct mapping of the terminator atmosphere. Other approaches that explicitly separate out the contributions from the morning/evening limbs during ingress/egress have also been developed and applied to the highest signal-to-noise targets from JWST (Espinoza et al. 2024; Fu et al. 2025).

#### Stellar heterogeneities

We have discussed the impact and inclusion of stellar activity representation in the model for retrievals, but often corrections are also required at the lightcurve fitting stage. Modeling approaches to dealing with stellar activity are primarily intended to account for unocculted spots and faculae, which do not have a clear signature in the lightcurve. Conversely, spots or faculae that lie on the transit chord are occulted, and this will often produce a bump or a dip in the lightcurve at the point of occultation (see Fig. 7). Early best practice in the event of occulted starspots would be to remove the section of lightcurve containing the spot anomaly, and then fit the rest of the lightcurve to extract the transit parameters (e.g, Sanchis-Ojeda et al. 2011). However, this approach removes information about the spots. More recently, tools have been developed, such as PyTranSpot (Juvan et al. 2018), which enables multi-band lightcurve modeling of spots and transits simultaneously. This provides the spot contrast as a function of wavelength, which is informative about the spot temperature relative to the unspotted photosphere.

For smaller, cooler stars in particular, flares can also present a challenge to lightcurve fitting. TRAPPIST-1 is an excellent example of this, with multiple flares seen in transits of the TRAPPIST planets captured with JWST. Howard et al. (2023) present spectroscopic observations of flare-contaminated transits of TRAPPIST-1 b, f and g. They fit for the transit and extract the flare spectra in order to study the characteristics. TRAPPIST is a useful benchmark system for such analyses with a wealth of data and multiple observed transits of each planet providing good prior constraints on the planetary transits, allowing the competing effects to be disentangled at lightcurve level. Understanding flares on these stars is crucial for appropriate corrections in contaminated transits.

The treatment of stellar limb darkening is also crucial for lightcurve fitting. This is parameterized in the lightcurve fit, following one of a variety of possible prescriptions, e.g. as described by Sing (2010) and Espinoza and Jordán (2016). Various computational tools exist for calculating limb darkening coefficients such as LDTK (Parviainen and Aigrain 2015) and ExoTiC-LD (Grant and Wakeford 2024). Recovering limb darkening coefficients from transit fits is not always straightforward, with challenges occurring particularly for high impact parameter transits (e.g. for L98-59 d, Gressier et al. 2024). If the planet transits at high stellar latitudes, the transit lightcurve samples only a small range of emission angles from the star’s surface, limiting the characterization of the limb darkening. This can in turn lead to greater uncertainty on spectroscopic lightcurves of the planet, since limb darkening may not be appropriately accounted for.

Finally, accounting for stellar chromospheric emission in lightcurve fitting is also shown to be necessary. Perdelwitz et al. (2025) reanalyze the transit of hot Jupiter HAT-P-18b across its active K dwarf host star, accounting for the line emission from the optically thin chromosphere. The resultant transit spectrum is overall similar in shape, but not identical, to a version using a more standard lightcurve fitting tool that does not include chromospheric emission. The apparently small differences in the spectrum have a substantial impact on results from spectral retrieval, with the retrieved limb temperature increasing by ∼200 K to be more in line with expectations based on the equilibrium temperature.

### Direct imaging and brown dwarfs

Whilst the majority of this paper aims to summarize the challenges for retrievals of transiting planets, it is in possible to obtain direct spectra for young substellar or planetary mass companions at wide separations from their parent stars (e.g., Goldman et al. 2010; Gauza et al. 2015; Marois et al. 2008; Gauza et al. 2015; Macintosh et al. 2015) and also to perform direct spectroscopy for isolated brown dwarfs (e.g., Kirkpatrick 2005; Cushing et al. 2011; Kirkpatrick et al. 2012). Recent examples of observations with JWST include a 1–20 μm spectrum of VHS 1256–1257 b (Miles et al. 2023), NIRCam integral field unit observations of all 4 planets in the HR 8799 system (Xuan et al. 2026), and a NIRSpec PRISM spectrum revealing auroral emission on binary brown dwarf W1935 (Faherty et al. 2024). These objects are not tidally locked and therefore lack the dramatic global temperature gradients that are a classic feature of transiting hot Jupiters. Instead, for these more isolated worlds the internal temperature is the main driver of atmospheric structure and composition. Observations of these young planets and brown dwarfs are analogous in terms of observation geometry to eclipse observations of transiting planets.

There are several examples of retrieval studies for brown dwarfs and directly-imaged companions based on ground-based and space-based data (e.g., Line et al. 2015; Mollière et al. 2020; Whiteford et al. 2023; Nasedkin et al. 2024b; Lueber et al. 2026). One key difference in retrievals for directly imaged objects from transiting planets is that there is no independent constraint on the radius of the planet. Radius is to an extent degenerate with the temperature of the object, which both impact the overall luminosity. Historically, for brown dwarfs this was further complicated by uncertainty on the distance to the object, since for field brown dwarfs there is no companion star to facilitate a well-determined distance; more recently, parallax measurements for brown dwarfs have improved. There is also typically no independent mass constraint, since most directly imaged systems are less amenable to radial velocity measurements, and for field brown dwarfs this is not possible anyway. In their retrievals of benchmark brown dwarfs, Line et al. (2015) retrieve a scaling factor (*R*/*D*)$^2$, where *R* is the radius of the target and *D* the distance. For their particular targets, the distance is known as both objects have stellar companions and so the radius can be directly inferred, but for other cases this approach could mitigate any radius-distance degeneracy. As is done for stellar models, the gravity log(*g*) of the target is also a free parameter in the retrieval, and if the distance is known then the radius and the mass can both be inferred from the directly retrieved quantities.

Some brown dwarfs and young giant planets show evidence for absorption by clouds, the properties of which can be recovered via spectral retrieval (e.g., Line et al. 2017; Mollière et al. 2020). Appropriate parameterization of clouds is therefore a key modelling step for these objects; e.g. Burningham et al. (2021) introduced a retrieval framework able to recover properties of Mie scattering clouds formed from specific condensates such as quartz and enstatite using look-up tables, before this was implemented for transiting exoplanet retrievals. Accurate line data is also very important, as shown by the dramatic improvement in model fits to brown dwarf spectra with the advent of a more complete methane linelist (Yurchenko et al. 2014), which has in turn been superseded by Hargreaves et al. (2020)

Whilst the large, longitudinally-varying temperature structures induced by intense irradiation on transiting planets are not present on these widely separated and isolated worlds, there is evidence for variation and patchiness in cloud cover (e.g., Radigan 2014; Crossfield et al. 2014; Metchev et al. 2015; Vos et al. 2023). This needs to be parameterized in retrieval frameworks in much the same way as partial terminator cloud coverage is included for transit spectra, with some fraction of the visible disc covered by regions with different cloud properties and the overall atmospheric signal is a linear sum of these. Unlike the typical transit case, the regions are not constrained to be a mixture of cloudy and clear, but instead be different combinations of cloud decks. Broad wavelength coverage is instrumental in providing sufficient information to constrain these more complicated cloud models (Vos et al. 2023). Time-dependent retrieval frameworks have recently been developed to infer mechanisms for observed rotational variability (e.g., Nasedkin et al. 2025; Wang et al. 2026), revealing in some cases that longitudinal variation in cloud coverage is not always the culprit and that drivers may instead be thermal or magnetic (Nasedkin et al. 2025).

Very precise, broad wavelength spectra also provide tighter constraint on the temperature structure of brown dwarfs and directly imaged planets than are generally available for transiting hot Jupiters, where the shortest wavelength data points that probe the deep atmosphere are relatively low contrast due to the star’s spectrum peaking at shorter wavelengths. Since our prior knowledge of brown dwarf atmospheres is better informed than that of exoplanets due to a longer observational history, it may be feasible in some cases where the data are of very high quality to revert to the much more efficient optimal estimation approach.

In summary, whilst there are certain differences between retrievals of brown dwarfs/young self-luminous planets and transiting planets, the challenges inherent in retrieval are broadly similar since many of these come down to the details of modelling substellar atmospheric properties. In the next section, we look at the data available and discuss how these affect the computational approaches we must take in retrieval.

### High-resolution spectroscopy

The previous section describes modelling challenges specific to retrievals from low- to moderate-resolution spectra. Alternative techniques exist for characterisation of exoplanet atmospheres using ground-based high resolution spectroscopy, for which retrievals can also be used. High resolution (R∼100,000) cross correlation techniques rely on the known Doppler shift of the planet signal due to its orbit, which causes the planet’s absorption lines to slide across the stellar and telluric spectrum with time. If an appropriate atmospheric spectrum (i.e., template spectrum) containing the absorbing species is cross-correlated with the processed data, the summed similarity between the template and the observations will produce a peak at the planet’s expected velocity if the species is indeed present (Snellen et al. 2010).

From a data perspective, the main challenge for these high-dispersion spectroscopic data is the heavy processing required to remove telluric lines, stellar signals, and instrumental systematics. A side-effect of these post-processing steps is attenuation and/or distortion of the planetary signal itself. Some of the most commonly used methods for suppressing telluric and instrumental systematics, while aiming to preserve the time-variable planetary signature, are SYSREM, Principal Component Analysis and Single Value Decomposition (e.g., Birkby et al. 2013; Tamuz et al. 2005; de Kok et al. 2013; Brogi and Line 2019; Gibson et al. 2022). While the specifics of each method vary, the objective is to identify and remove dominant and coherent structures in the data leaving behind the planetary signal only. These approaches are effective for isolating the Doppler-shifted planetary lines, but they also modify the variance structure of the data and attenuate the underlying planet signal. A direct consequence is that estimating the effective uncertainty on each datum becomes challenging: once the spectra have been normalized, detrended, and projected into basis vectors, the original noise properties no longer apply.

High-resolution spectroscopy quickly became a powerful technique for inferring the presence of atmospheric absorbers in exoplanetary atmospheres. However, given that the continuum is usually removed or normalised out of the spectrum, abundance determinations were historically considered challenging or impossible. This is due to the fact that the information about abundance largely comes from the contrast between absorption-line depths and the continuum level of the spectrum. In principle, observations providing access to a larger set of spectral lines with differing intrinsic strengths might partially help break degeneracies, but the fundamental limitation remains that the continuum information would have been removed. The assumption that abundances could not be retrieved from high-resolution spectra was ultimately overturned by adopting a likelihood framework that operates directly on the processed residual spectra and accounts for signal attenuation and modified noise properties (e.g., Brogi and Line 2019; Gibson et al. 2020), as further explained in Sect. 5.2.2.

## From priors to posteriors using parameter estimation

As already introduced in Sect. 2, a ‘retrieval’ is essentially a problem of parameter estimation; the process of inferring the values of the parameters in a model ($\mathcal {M}$, Sect. 3) using a set of data ($\mathcal {D}$, Sect. 4). While an entire branch of statistics is devoted to estimation theory, here we focus on the probabilistic approach and briefly discuss both frequentist parameter estimation and Bayesian parameter estimation. At their core, both methods fit a model to data to find the parameter values that ‘best’ explain the observations. Their distinction lies in how probability is interpreted, how prior knowledge and beliefs are incorporated, and how uncertainty is represented. In what follows, we describe how these data-model inference frameworks operate in the context of ‘*atmospheric retrievals*’.

### The algorithm

The computational efficiency of a retrieval is limited by two factors: the cost of a model evaluation and the algorithm used to explore the parameter space and evaluate the ’goodness-of-fit’. Over the last two decades the advances in both the model complexity (discussed in Sect. 3) and the algorithm used to perform the parameter estimation have led to the evolution and wide-adoption of retrieval techniques.

Within the context of exoplanetary observations, parameter estimation started at its simplest level by performing a brute-force search of a large grid of model evaluations. Madhusudhan and Seager (2009), for example, explored a grid of $\sim 10^7$ model evaluations spanning key parameters of interest (e.g., temperature, molecular abundances) using the weighted mean squared error (i.e., $\chi ^2$ divided by the number of data points) as the goodness-of-fit statistic. Such grid-search mapped some of the correlations between the model parameters but failed to deliver a statistically principled characterization of parameter uncertainties or degeneracies. When the model is inexpensive to evaluate, as in semi-analytical formulations, simple nonlinear least-squares optimization has also been used to locate the maximum-likelihood estimator (MLE, e.g., Heng and Kitzmann 2017). These approaches identify a single optimal parameter vector but do not provide posterior distributions.

In contrast, atmospheric remote sensing in the Solar System has long relied on statistically grounded inversion methods (e.g., Rodgers 2000). There, abundant complementary information–such as in situ measurements or well-characterized prior profiles supports the use of Optimal Estimation (OE). OE solves a Bayesian inverse problem but imposes Gaussian prior and error assumptions. For Solar System applications, where prior knowledge is strong and data quality is high, such assumptions are often justified. For exoplanet atmospheres, however, the combination of weak priors and low signal-to-noise makes these assumptions restrictive: OE can become strongly prior-dominated, and the Gaussian approximation can fail to capture the typical non-Gaussian or bounded posteriors.

OE was also adopted in the early stages of exoplanet atmospheric characterization, and several studies applied it directly to HST and Spitzer observations. In cases where the data is sufficiently informative, OE produced stable solutions and provided a useful bridge between Solar System retrieval practice and exoplanet applications. However, as exoplanet spectra at the time were generally low-signal, sparse, and often had poorly constrained key parameters, OE frequently became prior-dominated, and the Gaussian approximation failed to capture the strongly non-Gaussian or bounded posterior shapes typical of exoplanet spectroscopy studies. These challenges did not render OE unusable, but they highlighted the need for fully Bayesian sampling methods that do not impose restrictive assumptions on posterior shape or prior structure.

This transition naturally motivated the broader adoption of flexible probabilistic algorithms — most prominently Markov Chain Monte Carlo (MCMC) and later Nested Sampling (NS) — for exoplanet retrievals. MCMC methods approximate the full posterior distribution by evolving chains of walkers through parameter space, relaxing the Gaussianity assumptions. In principle, these methods provide full posterior distributions and parameter covariances. In practice, however, standard MCMC methods can struggle with multimodal, highly skewed, or high-dimensional posteriors. Furthermore, assessing convergence can be challenging and the method overall can be computationally expensive.

To address these issues, the field increasingly turned to Nested Sampling (NS). Originally developed in statistics and now central to cosmological parameter inference, NS explores parameter space by evolving a population of “live points” that progressively contracts around nested likelihood contours. Conceptually similar to MCMC in that it maps the posterior distribution, NS offers two key advantages. First, its acceptance–rejection structure enables efficient exploration of complicated or multi-modal posteriors, often in dimensions of order 20–30. Importantly the dimensionality of the atmospheric model (i.e., the number of model parameters) is a limiting factor since the computational cost for NS algorithms scales as $\mathcal {O}(\textrm{d}^3)$ (Handley et al. 2015). Second, and critically, NS provides a numerical estimate of the Bayesian evidence, the marginal likelihood obtained by integrating the likelihood over the prior volume. While the posterior samples are a by-product, the evidence calculation is the primary objective of nested sampling. With both the posterior and the evidence in hand, one can quantify parameter uncertainties and perform model comparisons.

Popular NS implementations include MultiNest and PyMultiNest (Feroz and Hobson 2008; Feroz et al. 2009, 2013; Buchner et al. 2014), UltraNest (Buchner 2021), and DYNESTY (Speagle 2020). While powerful and widely adopted, recent work has emphasized the need for careful configuration of the NS algorithm. For example, Dittmann (2024) demonstrated that insufficient numbers of live points or overly aggressive sampling efficiencies can bias evidence estimates and distort posterior distributions. They recommend running convergence tests across sampler settings, or using NS primarily to initialize an MCMC run when one is interested in posterior characterization rather than evidence evaluation. More broadly, the advent of NS makes it essential for practitioners to distinguish whether their primary goal is a robust posterior distribution or an accurate marginalized likelihood, as these objectives may demand different and sometimes mutually incompatible sampler configurations.

Overall, the transition from grid searches to OE, then to MCMC and Nested Sampling, reflects the increasing ambition and complexity of exoplanet atmospheric inference. Modern retrieval frameworks now routinely compute $10^4$–$10^7$ forward models per dataset. For analysis of panchromatic JWST observations of WASP–39b, individual retrievals have required up to $10^8$ model evaluations to robustly characterize posterior structure and enable model comparison.

#### Machine learning

Supervised machine learning (ML, e.g., Márquez-Neila et al. 2018; Zingales and Waldmann 2018) has been proposed both as a computational accelerator for atmospheric retrievals and as an alternative route to parameter estimation. In the simplest approach one generates a large training set of synthetic spectra spanning the prior (or a physically motivated region of parameter space), trains a regressor (e.g., Random Forest, deep neural network) to predict model parameters from spectra, and then applies the trained model to observed data. The principal appeal is speed: once trained, a model can make predictions orders of magnitude faster than an on-the-fly sampler because the expensive forward runs used to create the training set need not be repeated for each observation. In this sense, ML aims to amortize the cost of retrieval by shifting the computational burden to a single, upfront training phase.

A detailed comparison between these ML approaches and the more traditional nested sampling methods was presented by Nixon and Madhusudhan (2020). Their work reproduces previous Random Forest implementations for atmospheric retrievals (e.g., Márquez-Neila et al. 2018) and demonstrated that the ML method can reproduce nested-sampling posteriors for low-dimensional problems. However, they also showed that the computational advantage of ML degrades rapidly as the number of free parameters increases, because the training set must densely sample the underlying parameter space. In practice, ML retrievals can outperform nested sampling only at low dimensionality or in settings where a single amortized training set can be reused across many observations (e.g., population-level studies as may be the case for the Ariel mission). For commonly utilized, high-dimensional retrievals where tens of parameters may be required to describe a full atmospheric model, the training cost becomes prohibitive and standard Bayesian samplers remain more efficient.

Given that the purpose of a retrieval exercise is to quantify the range of possible solutions that can fit the data, it is paramount to understand uncertainty quantification from ML — a nontrivial quest. Naive histograms from an ensemble of trees (see e.g., Márquez-Neila et al. 2018) are not directly a Bayesian posterior and the uncertainties (i.e., percentiles) can therefore be under- or -over estimated. More fundamentally, standard ML regressors do not generally represent epistemic uncertainty, e.g., the uncertainty arising from limited training data or model misspecification, making it difficult to assess when the ML algorithm is evaluating spectra in regions of parameter space not covered by the training examples. Care must be exercised when building and interpreting the results from these ML algorithms (or any inference approach for that matter). Nonetheless, other approaches such as amortized inferences, emulators, and hybrid strategies combining ML-based proposals and MCMC/NS methods (e.g., Himes et al. 2022) offer promising avenues for achieving substantial speed-ups while retaining the principled uncertainty quantification of traditional Bayesian retrievals.

### The likelihood function

To measure the quality of the match between the model and the data a metric must be defined: the likelihood. Although the mathematical form is the same, its conceptual role differs slightly between the frequentist and Bayesian approaches. In a frequentist treatment, where the data ($\mathcal {D}$) are viewed as random realizations and the model parameters ($\theta _{\mathcal {M}}$) are fixed but unknown, the likelihood is a tool for estimating the parameter values that best explain the data — yet does not assign probabilities to those parameters. On the other hand, in a Bayesian treatment, the parameters have an associated prior distribution that quantifies prior knowledge or assumptions (further discussed below), and the data update these prior beliefs through Bayes’ theorem to yield a posterior distribution for the parameters.

For the remainder of this section we adopt the Bayesian interpretation, where the likelihood is the component in Bayes’ theorem that encodes our assumptions about how the data are generated given our model of the signal (i.e., astrophysical system) and the noise (i.e., any unknown or unwanted changes affecting the signal).

Following from the discussion in Sect. 4, the common assumption for spectroscopic observations of exoplanetary atmospheres, and in many fields of signal processing, is that they are independently distributed with Gaussian errors (e.g., ‘white noise’) and their probability density function is a normal distribution. Therefore, the probability of the data given the model under the assumption of independence between all *N* data points is

8 $$\begin{aligned} \mathcal {L}\left( \mathcal {D}\mid \theta _\mathcal {M} \, , \mathcal {M}_i \right) = \prod _{k}^{N} \frac{1}{\sqrt{2\pi }\sigma _{k}} \mathrm {\exp }\left( -\frac{[\mathcal {D}_{k} - \mathcal {M}_{i,k}]^2}{2\sigma _{k}^2}\right) , \end{aligned}$$

which is $$\propto \exp (-\chi ^2/2)$$.

While early results with JWST suggest that the data processing steps described in Sect. 4 deliver uncorrelated observations (e.g., Rustamkulov et al. 2023), this assumption embedded into the likelihood function has been previously explored within the context of the parameter estimation problem for exoplanetary atmospheres. Additionally, estimating the noise properties of data after it has been processed can result in complications for directly implementing Eq. (8) as in the case of ground-based high-resolution spectra of exoplanets which undergo scalings, shifts, and signal removal. We briefly discuss such cases below.

#### The treatment of noise: error inflation and correlation

Within the first few years of spectroscopic observations of sub-stellar atmospheres being analyzed using atmospheric retrievals, practitioners began exploring whether the reported precision on the observations was reliable. For instance, (Barstow et al. 2013) considered whether the reported error bars in the spectroscopic data of GJ 1214b had to be increased in order to enable the combination of observations taken at different epochs from different space- and ground-based observatories, that could be affected by temporal changes in stellar activity, different instrumental systematics, and data processing techniques. Other studies formally implemented changes to the likelihood function and included a term to increase the standard error on the data. This more formal approach allowed for the marginalization of this inflation parameter over all other relevant parameters in the atmospheric model.

There are diferent mechanisms for calculating the inflated error. Line et al. (2015) add an additional noise term in quadrature with the reported measurement error σ, with the total noise expressed by

9 $$\begin{aligned} \sigma '=\sqrt{\sigma ^2 + 10^b} \end{aligned}$$

where *b* is a free parameter in the retrieval. This adds a wavelength-independent noise term. As an alternative, Piette and Madhusudhan (2020) and Welbanks and Madhusudhan (2021) implement the approach from Foreman-Mackey et al. (2013) in which a wavelength-dependent term $x^2{f_m}^2$, where *x* is a fractional scaling on the wavelength-dependent value of the model spectrum realization $f_m$, is added in quadrature. Error inflation approaches have already been implemented when analyzing spectra of transiting exoplanets (e.g., Colón et al. 2020; Bell et al. 2024).

Nasedkin et al. (2023) perform injection-recovery tests on archival SPHERE and GPI data, to optimise image processing algorithms for directly imaged planets. They find that including the full covariance matrix in the likelihood calculation, rather than simply assuming uncorrelated noise, improves the accuracy of inferred parameters. This suggests that correlated noise is important for high contrast direct imaging, and this may of course extend to other types of exoplanet observation.

The likelihood function has also been modified within the context of grid-searches and high-precision brown-dwarf spectra as in the work of Zhang et al. (2020, 2021). These works adopt software tools, originally developed for the interpretation of stellar spectra, which incorporate non-parametric methods for Bayesian inference known as Gaussian processes (GP, Czekala et al. 2015). The goal of using these more flexible likelihoods is to account for possible data-model mismatches arising from model imperfections (e.g., limitations in the line-lists for atomic/molecular absorbers) and to capture possible correlations in the residuals. Briefly, a GP defines a covariance function (i.e., kernel) from which the full covariance matrix of the data is constructed and inserted directly in the likelihood function.

Beyond grid-searches, atmospheric retrievals computing the model evaluations during the parameter estimation have recently considered the use of GPs. For example, Aurora (Welbanks and Madhusudhan 2021) included publicly available libraries to evaluate the marginalized likelihood of a dataset under a GP model (e.g., Foreman-Mackey et al. 2017; Ambikasaran et al. 2015). More recently, Rotman et al. (2025) include these GPs as to model both global effects throughout the entire spectrum (i.e., a Global Kernel, e.g., error-inflation), and localized effects in one or more regions of the spectrum (i.e., Local Kernels, e.g., correlations in the data-model residuals). Their work using these non-parametric noise models to analyze synthetic and real JWST observations suggest that these more flexible methods can deliver less precise but more accurate constraints, especially in the presence of systematics and unknowns.

Taken together, these developments illustrate that departures from the idealized assumption of white, uncorrelated, and symmetric Gaussian noise can influence atmospheric inferences. For example, Davey et al. (2025b) used synthetic JWST datasets to demonstrate that strongly asymmetric observational uncertainties can bias retrieval results when modeled with a standard Gaussian likelihood, whereas adopting an explicitly asymmetric likelihood corrects much of this bias. More broadly, early JWST analyses (e.g., Carter et al. 2024; Holmberg and Madhusudhan 2023) highlight the importance of accounting for underestimated error bars and correlated residual structure when performing retrievals. Approaches such as error inflation, Gaussian-process covariance models, and flexible likelihood formulations therefore play an essential role by having a tradeoff between precision and robustness, reducing the risk of overconfident or misleading inferences driven by unmodeled systematics. The overarching lesson is that the likelihood function is itself a modeling choice, and its assumptions about noise correlation, variance, symmetry, and systematics shape the posterior as strongly as the forward model or the prior. Consequently, noise modeling must be treated as an integral component of the retrieval framework, with explicit justification and validation wherever possible.

#### A likelihood for high-resolution spectrography

When analyzing high-resolution observations, the likelihood function adopted is not fundamentally different from the low-resolution $\chi ^2$ likelihood; rather, the observable itself has been transformed by the data-processing pipeline, and the model must undergo the same transformation before the comparison can be made. After continuum normalization, detrending (e.g., SYSREM, PCA, SVD), and telluric/stellar removal, the information in the spectrum resides almost entirely in the pattern of absorption lines, not in their absolute flux. As a result, a direct, wavelength-by-wavelength flux comparison is no longer meaningful.

The high-resolution likelihood formulation introduced by Brogi and Line (2019) addresses this by expressing the Gaussian likelihood in terms of the projection of the model template onto the processed data, which mathematically becomes the cross-correlation between the two. Because the detrending removes the continuum and rescales the data, the noise variance cannot be taken from the instrument pipeline; it must be estimated from the processed data themselves. Solving for both the optimal template scaling and the noise variance yields a $\chi ^2$-like likelihood evaluated in this transformed space.

The key insight is therefore not that high-resolution data require a “new” likelihood, but that the same likelihood is applied after data and model have been processed in the same way. When this is done consistently, the cross-correlation amplitude becomes related to the gas abundance, because the strength and relative weighting of individual absorption lines depend on abundance. Using this framework, Brogi and Line (2019) showed that high-resolution spectra can constrain molecular abundances to precisions of order 0.5 dex. More flexible formulations of the likelihood, including explicit estimation of noise variance or modelling of correlated residuals, have been explored (Gibson et al. 2020), and have been applied to data obtained with instruments such as VLT/CRIRES, VLT/UVES, and GEMINI/IGRINS (Brogi and Line 2019; Gibson et al. 2020; Line et al. 2021).

#### Combining likelihood functions: low-res and hi-res

Once the likelihood is defined consistently for each dataset in its own observable space, combining low- and high-resolution information becomes conceptually straightforward: the total log-likelihood is simply the sum of the low-resolution likelihood and the high-resolution likelihood. This approach has been applied to the combination of ground-based high-resolution spectra and low-resolution spectra from HST (Kasper et al. 2023) and JWST (Smith et al. 2024). Take for instance the case of WASP-77Ab from Smith et al. (2024) who combined Gemini/IGRINS high-resolution (R∼45,000) eclipse spectroscopy with JWST/NIRSpec G395H observations. Their results suggest that their abundance constraints are dominated by the high-resolution data, because the cross-correlation likelihood is sensitive to detailed line-strength patterns, whereas the lower-resolution spectrum constrains the thermal structure and continuum level. The combination therefore leverages the strengths of each regime, yielding tighter constraints than either dataset alone.

More broadly, the ability to combine likelihoods reflects the underlying unity between low- and high-resolution retrievals: both are Gaussian likelihoods applied to data that have been transformed in different ways, and in each case the model must undergo the same transformations. The major practical difference is that high-resolution processing destroys the original per-pixel error estimates, requiring the noise properties to be inferred within the likelihood itself.

### The prior

Conceptually, a key difference between a Bayesian approach and a frequentist approach is the inclusion of a prior probability distribution for the parameters in the model. That is, Bayes’ theorem provides the framework to update a prior (e.g., what we think may be a reasonable range for the parameters in our model) with new data (i.e., the spectra) to obtain the posterior probability distribution (i.e., the answer we are after).

It may seem compelling to have the ability to have a prior belief which is then updated in light of new data. However, choosing a prior is non-trivial and in the presence of weak or low-signal-to-noise data, the resulting inferences can become strongly prior dominated. For example, we may expect most gas giant atmospheres to be H$_2$-dominated given results from interior-structure models or observations of the gas giants in our solar system. Alternatively, priors may be the result of subjective and arbitrary choices. For instance, what abundance for a given bio-signature should be considered reasonable in an exoplanet is highly subjective and up for debate given that, to date, we know of a single habitable planet. Finally, practitioners may adopt so called ‘uninformative priors’ aiming to assign equal probabilities to a wide range of possibilities. Nonetheless, no prior is truly uninformative; every choice imposes structure that will influence the posterior, sometimes substantially.

Within the context of sampling algorithms, the selected prior space is sampled to find the regions of high likelihood and deliver a posterior distribution. It is then evident that it is possible to select a prior that excludes some solutions even if they may be the ‘correct’ one. Conversely, as practitioners we may be compelled to choose ’wide’ and ’uninformative’ priors that allow for multiple scenarios to allow for the data to speak for itself and choose the correct scenario among all possible solutions. Nonetheless, in practice, even these wide priors can have limitations. For example, what should be the smallest possible amount of any given trace gas in an atmospheric model? Having an arbitrarily small number such as $10^{-12}$ may seem sufficiently small, but for some chemical absorbers like H$^{-}$ this abundance may be sufficient to produce noticeable features. Thus, even extremely small mixing ratios can materially influence the spectral fit, and lower bounds on abundances are not innocuous modeling decisions.

More broadly, exoplanet retrievals differ from Solar System atmospheric inversions in an important way: Solar System studies rely on decades of in-situ and laboratory constraints, allowing well-motivated and often Gaussian prior assumptions. Exoplanet studies lack such constraints, and the spectra are typically sparse and noisy, making the posterior far more sensitive to prior specification. This sensitivity is not a technical nuisance but a defining feature of the inference problem, and transparent documentation and testing of prior choices are essential.

On the issue of selecting a reasonable range of abundances for an atmospheric model, the field has adopted tools from compositional data analysis (e.g., Pearson 1897), specifically the use of a dirichlet distribution. In this approach, the priors for the different chemical absorbers in an atmosphere are chosen so that the unit-sum constraint is conserved in a way that is permutation invariant and the prior probabilities for all species is the the same. This approach is particularly helpful in cases where it is necessary to relax the assumption that the bulk composition of an atmosphere is a hydrogen-helium mixture as may be the case for sub-Neptunes and planets with secondary atmospheres. Here we are no longer assuming *a priori* that the atmosphere of the planet is Hydrogen rich and we are allowing for a wider range of compositions in our prior distribution.

This concept is formally known as the use of compositional parameters and was introduced to the field of exoplanet atmospheres by Benneke and Seager (2012) using MCMC. In this approach, the compositional parameters can have any value between -∞ and ∞, while the traditional volume mixing ratios have values between 0 and 1. However, implementing such a prior in nested sampling algorithms can be challenging due to the use of transformation between a unit cube (NS samplers construct samples by mapping points from a unit hypercube into parameter space; compositional priors require nonlinear transformations that alter volume elements and break the simple uniform–to–physical mapping assumed by many NS implementations), which saw a delay in the adoption of this approach given the rise of nested sampling tools in exoplanet retrievals. The implementation of these priors into nested sampling was presented by Welbanks and Madhusudhan (2021) and adopted in subsequent works (e.g. Damiano and Hu 2021; Piette et al. 2022). When combined with JWST data, this prior has enabled tantalizing suggestions of secondary atmospheres as in e.g. Banerjee et al. (2024) which revealed an atmosphere potentially dominated by SO$_2$ for sub-Neptune L98-59d.

Overall, prior specification is not an auxiliary detail but a core modeling choice in exoplanet atmospheric retrievals. Because the data often underconstrain the models, the prior can strongly shape the posterior and, in some cases, determine whether key atmospheric properties appear “detected", unconstrained, or entirely absent. Careful justification, sensitivity analysis, and transparency in reporting are therefore indispensable.

### The posterior

The posterior distribution represents the central outcome of a Bayesian retrieval; that is, the posterior is the quantity we seek. Conceptually, the posterior encodes the range of parameter values consistent with the data under the assumptions of the every component discussed above. However, the posterior must be interpreted for what it is: a conditional distribution and not an absolute statement about the true state of the atmosphere or the planet. It reflects what the combination of the data, the likelihood assumptions, the prior, and the chosen model family permits the sampler to infer.

A common misconception is to treat a sharply peaked posterior as evidence for a physical “detection". In practice, a narrow credible interval may result from the interaction of a restrictive prior, an overly simplified or ill-defined model, underestimated uncertainties, or an inflexible likelihood. Conversely, a broad or even effectively flat posterior does not imply that a parameter is unimportant; it simply indicates that the data, under the adopted assumptions, do not supply enough information to localize that parameter. Many key atmospheric quantities such as cloud properties, temperature gradients, or abundances of trace absorbers may regularly fall into this regime.

Posterior structure also carries information beyond its width. Multi-modality may indicate competing physical explanations that the data cannot distinguish; skewness or hard cutoffs often reflect prior boundaries or model non-identifiability; and degeneracies between parameters (e.g., reference radius–pressure, temperature–abundance) reveal fundamental limitations imposed by the data and the atmospheric model. As such, the posterior should be read as a diagnostic, not only a set of parameter estimates.

Finally, it is important to emphasize that the posterior does not evaluate whether the model itself is adequate. A well-behaved posterior can arise even when the model is incomplete or misspecified, and a poorly behaved posterior may simply reflect limitations of the data. Ultimately, the scientific insight comes from examining why an answer and the model it comes from succeeds or fails under particular assumptions, not from any single statistical quantity.

### The evidence

In addition to the posterior, Bayesian inference provides another quantity of interest: the marginal likelihood, or Bayesian evidence, defined as the likelihood averaged over the prior volume. While the posterior summarizes what parameter values are supported under a given model, the evidence quantifies how well the model as a whole explains the data under the combined assumptions of the likelihood, the model, and the prior space considered. It has become a key quantity in exoplanet studies due to it being readily available when using nested sampling algorithms.

The evidence is not required for parameter estimation and indeed, studies using MCMC for parameter estimation may not report it. Furthermore, the evidence does not measure “truth" or the detection of any quantity or parameter on its own. Instead, it is one of several tools available for model comparison (for an overview see, e.g., Welbanks et al. (2023)). A higher evidence indicates that, given the chosen priors, one model explains the data more efficiently than another. This makes the evidence a useful tool for model selection, but not a definitive arbiter. It remains sensitive to prior choices, model assumptions, and data limitations.

How, then, should these quantities be interpreted, and how should competing atmospheric models be compared in practice? We explore these questions in the following section.

## From posteriors to perspective—model interpretation and comparison

Having described how retrievals estimate parameters and produce posteriors, we now turn to how those posteriors are interpreted; how models are compared, how detections are claimed (or misclaimed), and how the boundaries between inference and interpretation often blur.

### Why interpretation is hard

Retrievals yield conditional statements about model–data consistency, not absolute truths. The interpretation of posteriors depends critically on model assumptions, prior structure, and data quality. Here we distinguish between parameter estimation and model selection. A detailed discussion of model comparison techniques within the context of retrievals of exoplanet atmospheres is available in Welbanks et al. (2023), while Welbanks et al. (2026) present an overview of the pitfalls in interpreting spectra in the era of JWST.

### Model comparison using Bayesian evidence

As explained in Sect. 5.5 the marginal likelihood, or evidence, is a single-value metric that is commonly used in exoplanet atmospheric retrievals to compare models. The evidence is, by definition, dependent on the assumed prior volume for the model’s parameters—which can be ill-defined or not fully physically motivated. The ratio of evidences for two models defines an associated Bayes factor which can then be represented as an odds ratio for the preference for one model over another.

While other fields of astronomy like cosmology have adopted Bayes factors as a way to quantify the preference for one hypothesis/model over another, the exoplanetary field adopted the notion of these model preferences as “detection significances”. This misnomer has resulted in reports of “detections” of chemical species, in many cases of no statistical significance and that do not correspond to detections in the frequentist sense (e.g., Madhusudhan et al. 2025), instead of specific model preferences that are relative in nature and do not imply the physical presence of a molecule in a planet’s atmosphere.

The use of Bayesian model comparisons using Bayes factors was proposed by Benneke and Seager (2013) as an approach to quantify the need for a particular chemical absorber in an atmospheric model under the assumption that: i) the hypothesis space is complete, e.g., the atmospheric model includes most of the expected absorbers for a planetary atmosphere, especially those degenerate with each-other; and ii) that the models considered are nested, e.g., the reference model is the one including the full-hypothesis space and it is being compared to the same model but excluding a single species of interest.

Procedurally, the Bayes factor is computed by running a retrieval using a model with, and then a model without, the species of interest included and then comparing the Bayesian evidence values for those runs. An increase in the Bayesian evidence when the parameter of interest is included means that its inclusion is preferred. The evidence thresholds introduced by Trotta (2008) are commonly adopted as a heuristic benchmark to determine the significance of the preference (e.g., the ‘detection’), with the log of the Bayes factor $\Delta \ln BE <1$ being inconclusive, 1 – 2.5 demonstrating weak evidence, 2.5 – 5 moderate evidence, and >5 strong evidence.

Bayes factors are sometimes converted to equivalent σ values to facilitate comparison with frequentist statistics, where 5σ is typically considered the gold standard for a detection. This conversion is performed by using the formula linking Bayes factors to p-values introduced by Sellke et al. (2001) and subsequently extended by Benneke and Seager (2013) to convert to σ.

The two assumptions underlying this framework have, in practice, been relaxed, with consequences for the interpretation of atmospheric spectra. First, the assumption of a complete hypothesis space is often weakened: rather than including a broad and physically motivated set of chemical absorbers, particularly those known to be spectrally degenerate, some studies (e.g., MacDonald and Madhusudhan 2017; Lewis et al. 2020) adopt a reduced set of species, which can artificially inflate apparent model preferences.

Second, while the use of nested models is generally maintained, the choice of reference (null) model has not been uniform across the literature. In the commonly adopted “remove-one-at-a-time” approach, the reference model includes a comprehensive set of absorbers and is compared to versions with individual species removed, thereby testing whether a given species is required beyond an otherwise adequate baseline. In contrast, Madhusudhan et al. (2025) adopt a minimal baseline model containing only two absorbers and assess evidence gains from adding additional species. Although the resulting model pairs remain formally nested, this alternative framing changes the null hypothesis being tested, from the necessity of a species to the improvement over an already simplified model, and consequently alters the interpretation of the resulting Bayes factors. Conditioning on a suboptimal reference model can amplify apparent “detection significance” without establishing the physical presence of the added species, as highlighted by Welbanks et al. (2026). We discuss these and other challenges in detail below.

### Challenges: common pitfalls in interpreting retrievals

Once a retrieval is complete, there are multiple challenges to its interpretation. The goal of retrieval is to determine the atmospheric composition, and be able to state the confidence with which we recover each gas. Of course, there is no such thing as a perfectly correct retrieval set up, and choices made during the retrieval process can impact our ability to accurately recover the correct atmospheric solution in different ways. We list some examples of pitfalls and challenges below.

#### Retrieval degeneracy

**Fig. 8:**
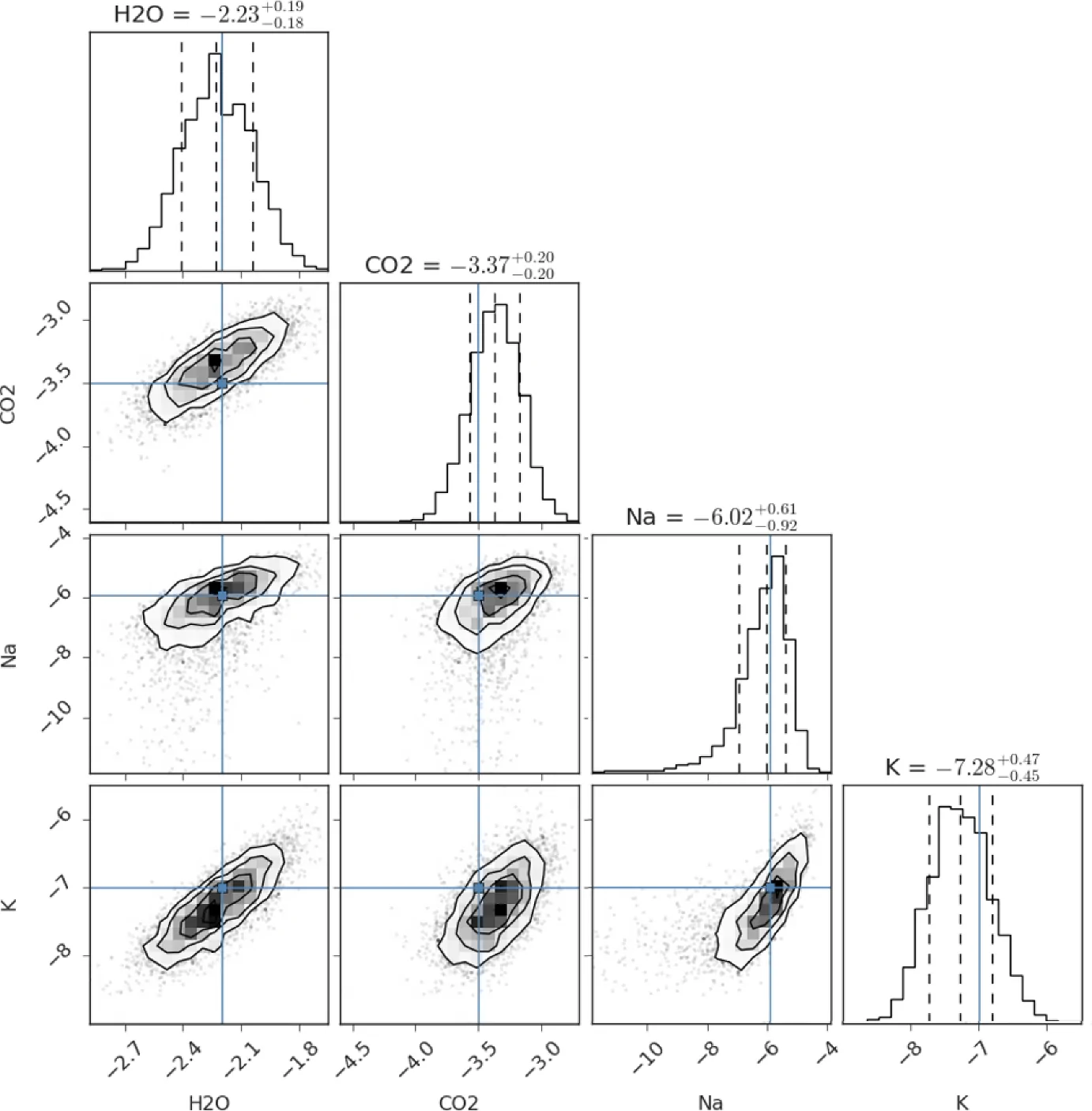
This figure shows the 1D and 2D probability distributions gas abundances retrieved from a synthetic spectrum of a hot Jupiter, with the input values indicated by blue crosshairs. The 2D histograms show clear positive correlation between the abundances. This is common, because the most spectrally dominant gas (here H$_2$O) sets the baseline for all other retrieved species

The shape of an exoplanet spectrum, whether transmission or thermal emission, depends on multiple factors, and it is often the case that the effect of changing one parameter can be mimicked by changing another, or a combination of others. This phenomenon is known as retrieval degeneracy (see Fig. 8). Typically, low signal to noise spectra or spectra covering only a narrow wavelength range are the most susceptible to this, but recent results have shown that even benchmark spectra such as the WASP-39b Early Release Science observation may have multiple possible solutions (Welbanks 2025). For WASP-39b, this is rooted in a fluke of its atmospheric composition. WASP-39b has a Saturn-like metallicity and a low C/O ratio, which results in a H$_2$O volume mixing ratio of around 1% of the atmosphere. At such quantities, the mean molecular weight of the atmosphere is non-trivially increased by the presence of H$_2$O. As the mean molecular weight increases, the scale height of the atmosphere decreases, which would reduce the size of absorption features in transit. Therefore, adding slightly more H$_2$O to the atmosphere reduces the scale height. However, adding more H$_2$O would also increase the absorption and have the opposite effect of *increasing* the feature size. The two effects of increasing mean molecular weight and increasing H$_2$O absorption very nearly cancel each other out, meaning that the likelihood function for H$_2$O abundance has a broad flat region for WASP-39b ranging a few % in H$_2$O abundance where changing the abundance has very little effect on the spectral fit. Therefore, despite exquisite data quality, the H$_2$O abundance cannot be constrained to the precision we would hope using free retrievals alone. The answer instead comes from a combination of free retrievals with more physically motivated modelling, including interior structure models and atmospheric grids (Welbanks 2025).

Breaking degeneracies can be challenging, especially as retrieval models become increasingly complex. Examples such as that given above for WASP-39b highlight the importance of a sanity check for retrieval results. This can be provided by post-retrieval comparison to results from alternative modelling frameworks (e.g., as in Welbanks 2025) comparison to results from interior structure models) or by inclusion of informative prior constraints within the retrieval itself (e.g. enforcing chemical equilibrium on the retrieved solutions.)

#### Retrieval bias

A perhaps more insidious problem is retrieval bias. Retrieval bias occurs when a particular modelling choice forces the retrieval to a solution other than the true one, and this can be introduced in many different ways. It is challenging to overcome largely because model fits to spectra can still be very good, so the solution may look plausible.

Several examples of model bias have emerged in recent years. For example, for ultrahot Jupiters, not accounting for variation in the temperature along the line of sight, or the dissociation of certain chemical species, can result in biases in the inferred atmospheric properties. Additionally, for cases in which the chemistry at either side of the terminator can be very different (e.g., ultrahot Jupiters) the chemical abundances recovered can also be biased towards the dayside composition (Caldas et al. 2019). However, the quality of fit to the spectrum is excellent for the biased retrievals and provides no indication that anything is amiss.

Another instance of model bias that can occur for large scale height planets with fast rotation periods is caused when models do not account for reduced gravity as a result of rotation. Whilst this is typically accounted for in solar system planets when the planet’s rotational period and gravity field are well known, for most exoplanet retrieval codes this is ignored. When a planet rotates rapidly, acceleration perpendicular to the rotation axis partially counteracts the force of gravity, as shown in Fig. 9 from Banerjee et al. (2023). A reduction in gravity will increase the scale height of the atmosphere, and the effect on the transit depth will be most signficant for planets where the scale height is already large. Banerjee et al. (2023) conduct a synthetic retrieval study on spectra generated using rotationally-reduced gravity, and find that for low density, hot planets in short period orbits (assuming tidal locking) that failing to account for the reduced gravity can bias gas abundances as the retrieval compensates for the increased scale height. They identify several JWST targets, including WASP-19b and WASP-121b, for which this effect is likely to be important.

**Fig. 9:**
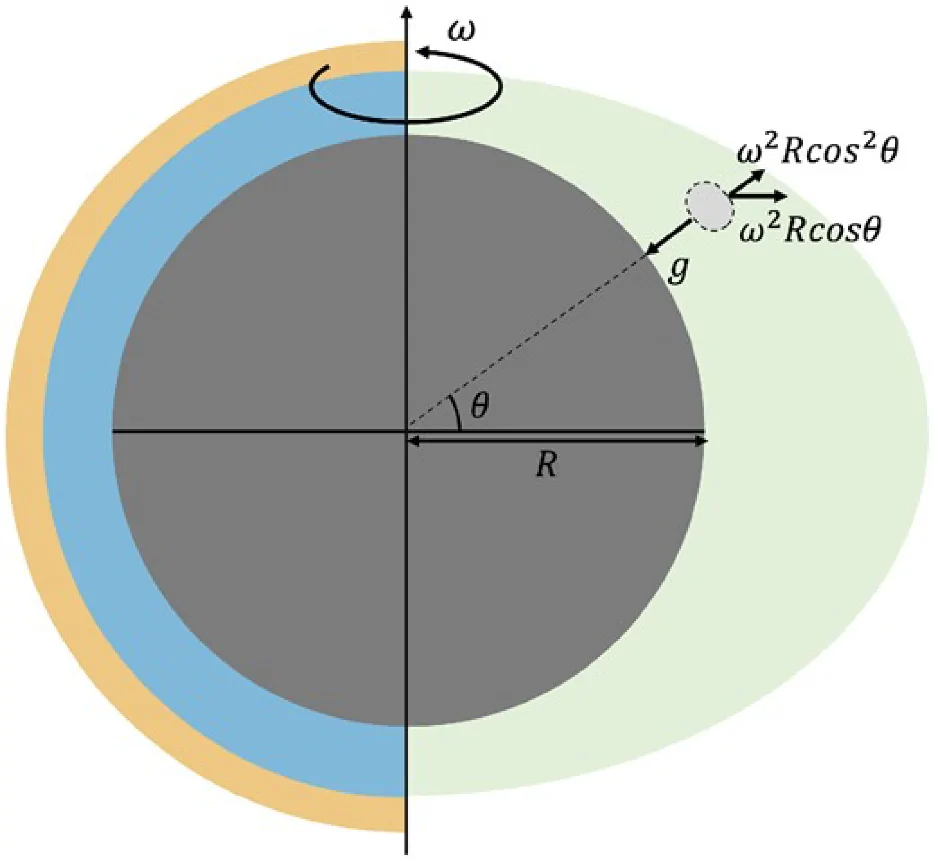
A schematic cross-section of a planet along the plane of the terminator showing the accelerations acting on an atmospheric parcel (light grey). On the left half of the diagram, the atmospheric area corresponding to the reduced gravity scenario is shown by an orange annulus (outer). The atmospheric area corresponding to the scenario without the rotation included is illustrated by a blue annulus (inner). On the right half of the diagram, an exaggerated elliptical atmospheric annulus is shown. The centrifugal acceleration acting on the parcel is perpendicular to the axis of rotation and marked as $\omega ^2 R\textrm{cos}\theta$, and the radial component of this acceleration which opposes the gravitational acceleration g is marked as $\omega ^2 R\textrm{cos}^2\theta$. Image reproduced with permission from Banerjee et al. (2023), copyright by the author(s)

#### Model completeness and evidence

As explained above, it is common practice to compare Bayesian evidences between different models and to interpret the resulting Bayes factors as “detection significances” for individual species, although in reality these are meant to represent *model-preference*. A recent preprint by Kipping and Benneke (2025) has highlighted additional concerns with how this procedure is implemented in the exoplanet literature, noting that the relation introduced by Sellke et al. (2001) may provide only an upper bound on the Bayes factor corresponding to a given p-value and therefore should not be interpreted as a direct conversion to frequentist significance. While this critique underscores the need for caution when translating Bayes factors into equivalent σ values, the more fundamental issue is that Bayes factors themselves are inherently relative quantities whose interpretation depends entirely on the chosen model set and reference hypothesis (Welbanks et al. 2026). Whether or not a conversion to σ is performed does not change this underlying limitation.

The quoted detection significance in any given study is therefore intrinsically valid only within the context of the models considered. A notable recent example is the claimed detection of dimethyl sulphide (DMS) and dimethyl disulphide (DMDS) in the atmosphere of the sub-Neptune K2-18 b (Madhusudhan et al. 2025). In that work, an atmospheric model containing either DMS, DMDS or both alongside CH$_4$, and CO$_2$ yields a higher Bayesian evidence than a model with CH$_4$, and CO$_2$ only. In this case, both models do not consider H$_2$O despite the possibility of K2-18 b being a so-called ‘water world’. Additionally, several hydrocarbon species with absorption features overlapping the same wavelength regions are not included in the models being compared.

Using the same JWST data for K2-18 b and adopting similarly restricted models, Welbanks et al. (2026) demonstrate that these modeling choices fundamentally limit interpretability. By fitting models containing only CH$_4$, CO$_2$, and a single additional hydrocarbon at a time, they show that multiple species not considered in the original analysis, such as C$_3$H$_4$. provide fits to the observed spectrum that are statistically indistinguishable from those obtained with DMS and DMDS or better. In this context, the reported ‘detection’ of DMS and/or DMDS quantifies preference only relative to a narrowly defined and incomplete model space. It does not constitute evidence for the physical presence of these molecules unless the broader chemical hypothesis space can be convincingly excluded. Model completeness is therefore a critical assumption when quoting detection significances.

Ultimately, Bayes factors should be interpreted as measures of relative model adequacy rather than as direct indicators of detection. This distinction becomes especially important for secondary atmospheres of small, cool planets, where chemical complexity is high and the true atmospheric composition may lie outside the tractable subset of models that can be explored. In such cases, caution is warranted when translating model preferences into claims about the presence of specific atmospheric species.

#### Alternative and complementary metrics

Even in the case where a model is appropriately complete, claims of detections should be approached with care. Another challenge that can arise here is the dependence of a detection on only a small fraction of the available data points. Welbanks et al. (2023) identify an example of this in the detection of H$^-$ absorption in the atmosphere of HAT-P-41b presented by Lewis et al. (2020). Welbanks et al. (2023) apply Bayesian leave-one-out (LOO) cross validation to this data. LOO cross-validation evaluates the predictive power of each observation by fitting the model repeatedly with each data point omitted in turn, allowing the identification of specific points that disproportionately drive model preference.

While exact LOO would require rerunning a retrieval for every individual data point and is therefore computationally prohibitive, Welbanks et al. (2023) employ the Pareto-smoothed importance sampling (PSIS-LOO) approximation (Vehtari et al. 2024, 2017), which enables this analysis using a single retrieval. They find that the apparent H$^-$ detection in HAT-P-41b is largely driven by a single photometric point at 3.6 μm from Spitzer. Subsequent JWST observations have demonstrated that baseline offsets between instruments observed at different epochs are common (e.g., Carter et al. 2024), making a detection that hinges on the relative depth of a single broadband point a clear warning sign. Consistent with this concern, the reported transit depth at 3.6 μm was revised in a later study (Sheppard et al. 2021).

**Fig. 10:**
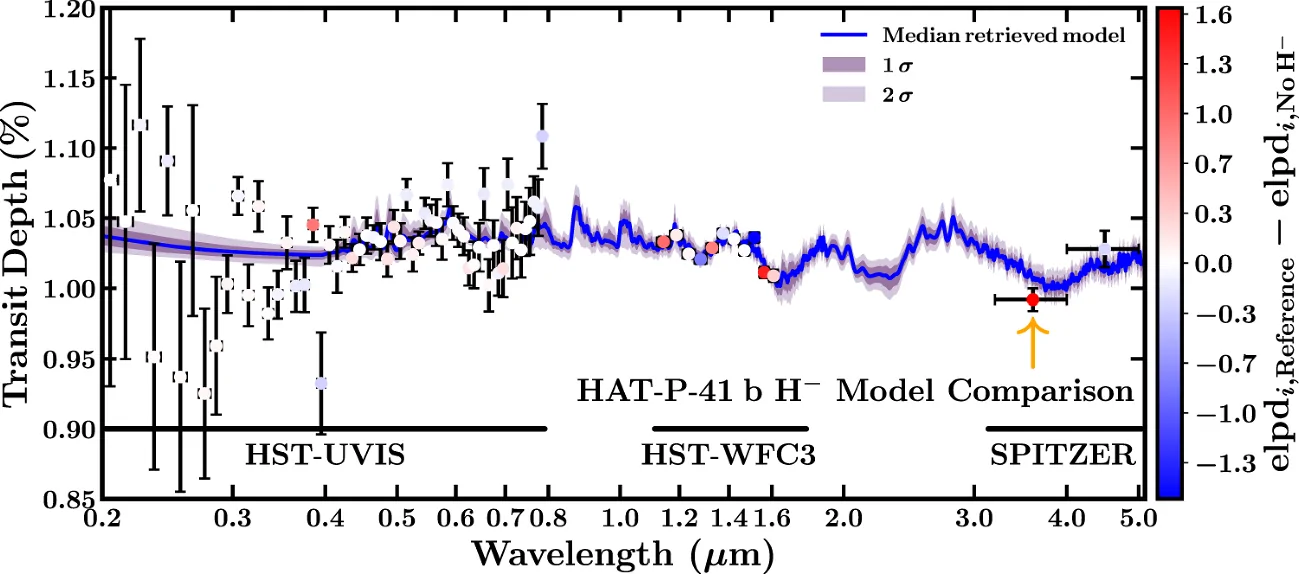
This figure shows the retrieved spectrum of HAT-P-41 b from HST UVIS G280 observations with systematic marginalization treatment, HST WFC3 G141, and Spitzer IRAC 3.6 μm and 4.5 μm observations from Wakeford et al. (2020) and Lewis et al. (2020). Data points are color-coded by the Δelpd score between models with and without H$^-$ absorption. Redder data points are better explained by the model with H$^-$ absorption while the bluer points are better explained by the model without. The orange arrow indicates the data point with the highest score (3.6 μm), which is a Spitzer photometric data point with an unknown baseline offset compared with the WFC3 G141 data. Image reproduced with permission from Welbanks et al. (2023), copyright by the author(s)

A complementary perspective is provided by recent work (Welbanks et al. 2024) that applies an analogous leave-feature-out approach to the inferred presence of NH$_3$ in WASP-107 b when analyzing its HST/WFC3, JWST/NIRCam, and JWST/MIRI spectra. For the leave-feature-out approach, the robustness of an inferred atmospheric signal is assessed by examining how densely and independently a given spectral feature is supported across wavelength space and whether the ‘detection’ depends more heavily on one instrument or another. Rather than asking whether a model performs better when a single molecule is included, this approach evaluates whether the inferred signal persists when subsets of the spectral features associated with that molecule are removed. Such analyses can be helpful to determine whether detections relying on a small number of isolated features or narrowly localized wavelength regions are particularly vulnerable to instrumental systematics, model mismatch, or coincidental spectral overlap (Fig. 10).

#### Challenges in hi-res retrievals

Even with dedicated retrieval frameworks and new instrumentation, challenges still arise in constraining the atmosphere using free retrieval approaches. Ultrahot Jupiters present a particular challenge (Brogi et al. 2023); molecules such as H$_2$O that are critical for constraining the overall metallicity and carbon to oxygen ratio for cooler planets are dissociated for the highest temperature objects, giving way to other absorbers such as H$^-$ to which high resolution observations are relatively insensitive. In this case, equilibrium chemistry retrievals or grid-retrieval approaches (where the likelihood function is computed by interpolating over a grid of pre-calculated models) may be more appropriate as this places additional prior constraints on the outcome of the retrieval.

The atmospheric modelling considerations for high spectral resolution retrievals are much the same as for lower resolution, with the exception of the treatment of absorption data. For high spectral resolution, correlated-k tables are generally inadequate and either a full line-by-line calculation or very high resolution cross-sections are used. Whilst at low and moderate resolutions the line strength precision is most important, for high resolution observations the line position is just as critical. For high temperature line databases generated from quantum mechanical simulations, ensuring accurate line positions requires refinement of the energy levels using empirical data (McKemmish et al. 2019), otherwise line positions may be sufficiently inaccurate such that cross-correlation analysis fails to detect a gas even though it is in reality present (e.g., Hoeijmakers et al. 2015; Merritt et al. 2020).

In the same way as for lower resolution retrievals, recent innovations have taken place towards retrieving spatial variation in atmospheric properties from high resolution data. Gandhi et al. (2022) introduce a framework, HyDRA-2D, that allows for separate constraints on the morning and evening terminators and day-night winds. This results in doubling the number of atmosphere parameters in the model, as each limb is treated independently; the wind speed parameters are assumed to be the same for both. This has been applied to ultrahot Jupiter WASP-76b, and preferred over a homogeneous model at >4σ significance. In this example, the total number of parameters for each limb was 10; the Fe abundance, six T-P parameters, and three cloud/haze parameters, leading to 23 free parameters in total across both limbs and with three windspeed parameters. For retrievals of a single species on a planet such as this where limb differences are expected, this approach seems well-justified; if simultaneously retrieving multiple gas abundances the parameter space may start to become unfeasibly large.

### Mitigation

Clearly, any chosen model will contain simplifications that fall short of the truth, but making choices to minimize the impact of these is a subtle but important step within the spectral retrieval process. This might mean running multiple retrieval tests with different models before embarking on a full analysis, or even simply comparing forward models initially to check that the model includes the necessary components. Chemical equilibrium simulations and other more physically-grounded models such as GCMs can also be helpful in identifying an appropriate starting point.

Erring on the side of caution, one may be tempted to include as many free parameters as possible as a first guess, and then reduce these on the basis of initial results. Whilst this is a good way of ensuring the initial retrieval is agnostic, large numbers of parameters also mean the retrieval will be slow and use a lot of computational resource. This was certainly the case in the extensive retrieval study of WASP-39b present by Welbanks (2025); however, this retrieval can now serve as a benchmark and starting point for retrievals of other, similar planets. Very extensive retrieval studies such as this are likely inevitable for the first targets observed with a new telescope or instrument, and represent an important learning step.

The inclusion of appropriate input data to the forward model is also of critical importance to a successful retrieval, as evidenced by biases introduced due to cross-section resolution, and detection challenges at high spectral resolution due to inaccurate line positions. Whilst producing these data are not a challenge for the retrieval practitioner per se, ensuring the retrieval model is using the latest version of a linelist or collision-induced absorption parameterization is important. The existence of accessible and well-maintained databases, notably ExoMol and HITRAN/HITEMP, greatly facilitates this, as do collaborative spaces created by meetings such as the ∼3-yearly ExoMol conference series. In addition, it is useful for retrieval publications to explicitly report where absorption data are unsuitable, inadequate or missing, as this is important feedback for the teams working on providing these data.

The quality of model inputs is constantly evolving in response to pressure from observational capability. As well as gas-phase absorption data, suitable scattering parameters for aerosols are becoming a particularly critical need in the JWST era, and this requires investment not just in computational facilities but also in laboratory experiments (e.g. He et al. 2020). The complexity of aerosol parameterization may also drive a need for machine learning approaches to linking aerosol composition and spectral characteristics.

## The future of retrievals

The last few years have demonstrated the need for rapid modeling innovation to keep pace with data from new observatories. This is particularly true where the emerging data reflect a real transformation in information content, which has certainly been the case for JWST. As new technology enables observation at different levels of precision, wavelengths, and geometries, the retrieval techniques we use will also have to evolve. Likely needs for increased model complexity will have to be tensioned against efficiency — for example, by improving our prior constraints through laboratory study or other types of modeling. Techniques for model comparison will also need to evolve, especially as we come closer to being able to study ‘Earth twin’ planets which may reveal hints of life outside the solar system. Our language and communication around detection will also need to be carefully re-evaluated in the light of this.

### Future observatories

**Fig. 11:**
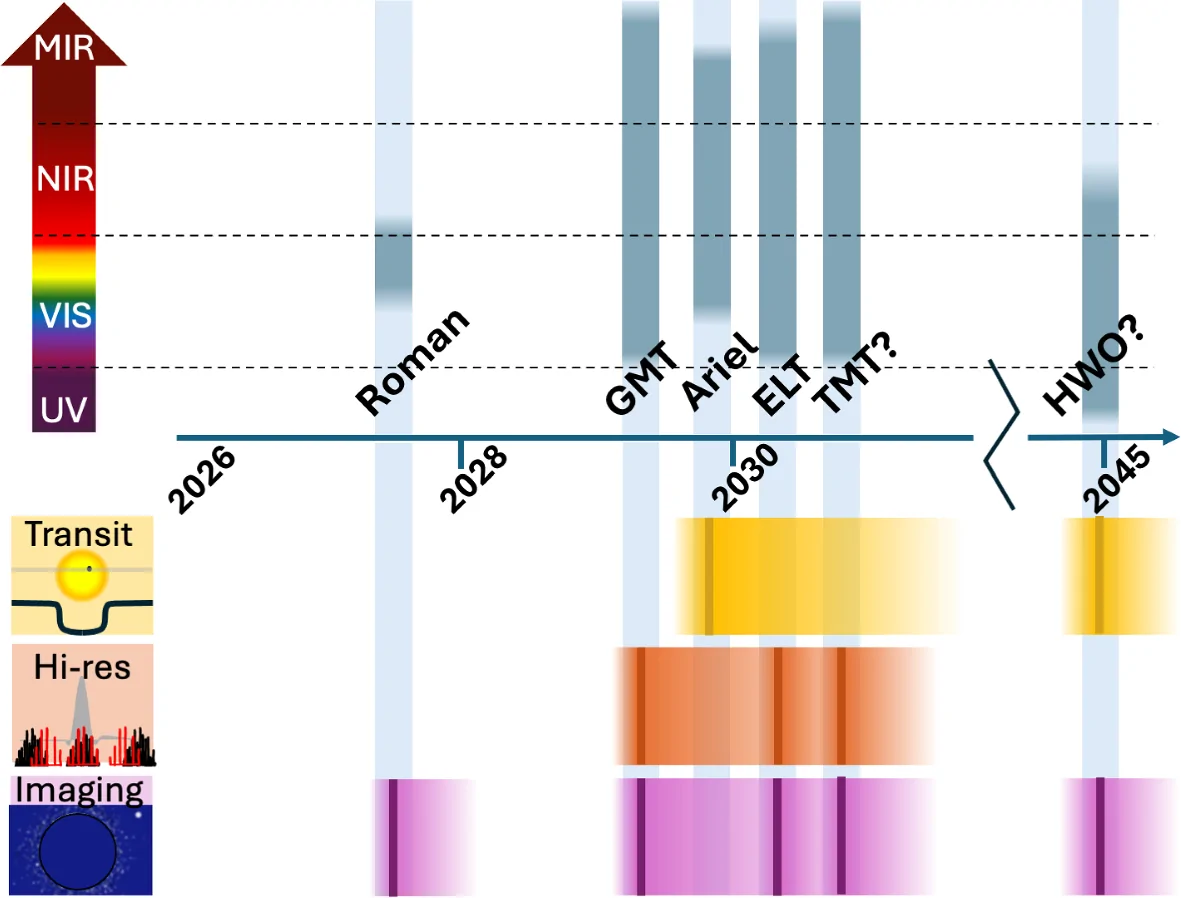
This schematic shows the dominant exoplanet observing modes and associated wavelength ranges to be accessed by future space and ground-based telescopes over the next two decades. First-light/launch timings for TMT (Thirty Meter Telescope) and HWO (Habitable Worlds Observatory) are currently uncertain. The three ground-based ELTs (Extremely Large Telescope, Giant Magellan Telescope and TMT) will all focus on high resolution spectroscopy and direct imaging, whereas space-based observations are more suited to transits. The three ground-based telescopes will also have their wavelength coverage limited by Earth’s atmospheric absorption. HWO will have coronagraphic imaging capability, and is the only planned observatory to access the near-ultraviolet

The main driver for increased retrieval model complexity is a dramatic increase in the information content of available data. At the moment, this is mostly driven by JWST (see e.g., Welbanks 2025), but in the near future we will also see dramatic improvements in high resolution spectroscopy and direct imaging from the ELTs. All of these observational advances will fuel the need for similar advances in modelling complexity, accuracy and efficiency (Fig. 11).

#### JWST

JWST is proving to be a powerful tool for the characterisation of exoplanet and substellar atmospheres, both for transiting exoplanets and directly-imaged companions. It has massively increased the wavelength access of space-based observations, as well as improving the precision and resolving power. This has in turn increased the information content available, resulting in several model simplifications that were previously reasonable now running the risk of generating spurious results.

We have already discussed above the requirement for spatial information to be encoded in retrieval frameworks, which is a direct consequence of JWST’s increased precision meaning that observations are actually sensitive to this information. The increased wavelength range available also means that a greater number of gasesous species must be included in models, and even for transmission spectra it is now the case that isothermal pressure-temperature profiles are inadequate, as stated above. All of these things lead to retrieval problems that have increasingly high dimensions, and are therefore more computationally intensive and also more at risk of failure/errors (see e.g., Dittmann 2024, discussed further in Sect.6.3).

Data at higher spectral resolving power that covers a broader wavelength range also requires a larger number of radiative transfer calculations, since this must be completed as a minimum at each spectral wavelength, and potentially at higher resolution before binning down. Taken together with the increased dimensionality of the problem, the typical computation time for a standard retrieval has increased to of order 10,000 core hours or more. This is especially challenging because it is generally considered necessary to run multiple retrieval instances within a single study—for example, a calculation of the evidence ratio for the presence of a particular gas generally relies on running retrievals with and without that gas included. It may also be deemed necessary to test a variety of cloud models and thermal profiles.

Therefore, whilst the data provided by JWST is undoubtedly transformational in terms of what we are able to learn, the computational burden it inevitably imposes is considerable, and it will clearly be necessary to optimise the process further in order to make the problem more tractable. One possible approach is to reduce the number of free parameters by placing more physical constraints on the retrieval (e.g. by running chemical (dis)equilibrium models), but as found by Al-Refaie et al. (2024) incorrect assumptions here can result in significant inaccuracies in the retrieved properties, despite an apparently reasonable fit to the data. Since JWST is providing access to information about these planets that we have not previously been able to access, it is also likely that the assumptions we have built on so-far limited data will require some refinement; a good example of this is the unforeseen detection of SO$_2$ on WASP-39b (JWST Transiting Exoplanet Community Early Release Science Team et al. 2023; Rustamkulov et al. 2023; Alderson et al. 2023). Replacing free parameters with physical constraints will undoubtedly save computation time, but quite possibly at the expense of accuracy since our knowledge of the processes at work on exoplanets is still very much a work in progress.

#### Ariel

The Ariel mission, due to launch in 2031, presents some challenges for spectral retrieval that are similar to JWST. Whilst the spectral resolution for Ariel is much lower than it is for several of the available JWST spectroscopic modes, the wavelength coverage is not dissimilar, and in fact all spectra will cover the wavelength range from 0.5–8 μm. Whilst the number of wavelength bins for the radiative transfer calculation is not as high as for JWST, the number of gas species that will need to be considered is equivalent. The precision expected is also high compared with what pre-JWST observatories were able to achieve, and for the best targets some of the more complex effects we are sensitive to with JWST will also be observable with Ariel.

The major challenge for Ariel, however, is the sheer number of planets it will characterize — 1000 at least in the space of a 3.5 year science mission. This is a much larger number than JWST is able to observe, and across those 1000 planets there will be a range of data quality and observational type (transits, eclipses, phase curves). A major effort within the Ariel team has therefore been the development of machine learning techniques, both for data reduction/cleaning purposes and for spectroscopic modelling and retrieval. This has taken the form of challenges advertised to the wider machine learning/AI community, which enables the team to make use of non-subject-specific expertise in useful techniques (see e.g., Hou Yip et al. 2022; Changeat and Yip 2023; Nikolaou et al. 2023; Aubin et al. 2023). The winners of the 2023 data challenge describe in Aubin et al. (2023) the use of Normalizing Flows trained on the results of synthetic spectra and corresponding Nested Sampling retrievals. They find that their algorithm is able to reproduce the same retrieved values, although it loses information about the correlation between parameters.

A significant advantage of a dedicated mission such as Ariel is the time available for exoplanet observations. This could potentially allow a larger fraction of the mission time to be spent on phase curves as opposed to transit and eclipses only. It is currently being suggested that up to 25% of the total mission lifetime could be dedicated to spectroscopic phase curve observations. This is supported by Valentine et al. (2025), who investigate the prospects for eclipse mapping with Ariel and compare likely constraints to those obtainable with JWST; they find that, whilst even with 20 Ariel eclipses of WASP-17b the hotspot offset precision of the JWST eclipse would not be matched, a single Ariel phase curve significantly improves on existing constraints from JWST. This drive towards more phase curve observations will likely elevate the priority of developing phase curve optimized retrieval frameworks, which can maximise the return on these observations.

#### ELTs

With the advent of ELTs, (Extremely Large Telescope, ELT; Giant Magellan Telescope, GMT; Thirty Meter Telescope, TMT) which will begin in the next decade, exoplanet spectroscopy is likely to begin a shift towards non-transiting targets, either via high-resolution spectroscopy or coronagraphic/high contrast imaging. First-light ELT instruments that are likely to be most relevant for exoplanets are ELT/METIS (Bowens et al. 2021), which will provide coronagraphic imaging and moderate resolution spectroscopy in the L, M and N bands (∼3–13 μm); ELT/HARMONI, which boasts a high-contrast adaptive optics mode covering 1.45 −2.45 μm at a range of spectral resolutions (Houllé et al. 2021; Vaughan et al. 2024); the GMT/Large Earth Finder instrument, a high resolution optical echelle spectrograph capable of cross-correlation spectroscopy for detection and characterization (Szentgyorgyi et al. 2016); GMT/Near-Infrared Spectrograph (NIRS), which is a moderate- to high-resolution spectrograph covering JHKLM bands (Jaffe et al. 2016); TMT/MODHIS, a high-resolution optical spectrograph that will enable cross-correlation (Dubey et al. 2025); and TMT/IRIS, a moderate-resolution imaging spectrograph covering JHK bands (Wright et al. 2014). These instruments open up the possibility of imaging cooler gas giants (e.g. METIS; Bowens et al. 2021), performing reflected light spectroscopy on nearby rocky planets such as Proxima Centauri b (Vaughan et al. 2024) and cross-correlation spectroscopy with much greater sensitivity that on currently available telescopes (e.g. Large Earth Finder). Instruments such as MODHIS will reach diffraction-limited spectral resolving powers of 100,000 covering the JHK bands simultaneously; compared to its pathfinder instrument on Gemini, IGRINS, this is a resolving power improvement of more than a factor 2 and a range expansion of 0.5 μm at shorter wavelengths.

Many of the retrieval challenges for the ELTs will therefore be similar to those already highlighted for directly imaged objects and high resolution spectroscopy; the key differences will be these highlighted increases in resolving power and wavelength range, which will mean additional computational burden within the forward model calculation, and an increase in the required precision of absorption data. In addition, the prospect of reflected light spectra will mean that full multiple scattering calculations become a necessity. This is explored in more detail in the following Sect. 7.1.4, which discusses future space missions operating at optical wavelengths.

#### Roman, HWO and LIFE

Whilst this is still a long way in the future at the time of writing, preparations are beginning for the next flagship space telescope, the Habitable Worlds Observatory. Although the details of what this telescope will look like are still under consideration, informed by the work of the START team and the working groups set up under its umbrella, the observatory will have at least a 6m mirror and cover wavelengths from the ultraviolet to the infrared, and will have coronographic capabilities that will allow direct imaging of Solar System analog planets.

The Nancy Grace Roman Space Telescope (Roman), due to launch in 2027, will have a 3-month coronagraphic imaging test survey during the first 18 months of operation. This survey is intended to be a pathfinder for HWO and inform subsequent technology development. The Roman coronagraph will access wavelengths from 0.5−0.8 μm at resolving power of 50, reaching a contrast of 10$^{-9}$.^1^ This will make it possible for Roman to image Jupiter-like exoplanets in reflected light. By contrast, HWO will operate over a wavelength range from the near-UV (possibly down to 100 nm) into the near-infrared (probably up to around 1.8 μm). The aim is to achieve a contrast ratio of 10$^{10}$, enabling direct reflected light detection and spectroscopy of Earth analog planets. The Roman coronagraph is capable of polarimetry, with a polarimetric instrument also being advocated for HWO (Gordon et al. 2023).

The new challenges to atmospheric modelling and retrievals that HWO will bring are largely related to the wavelength range over which it will operate, and the direct imaging geometry. For transiting exoplanets, however, which will still form a key part of its science case, a new opportunity will be UV/optical light phase curves and eclipses. Currently, our only UV capability comes from Hubble, and UV-optical eclipses are only feasible for a small number of planets. These measurements are also low resolution and low signal-to-noise. A move to higher resolution/higher precision UV spectroscopy will require the relevant opacity data at these wavelengths, especially photodissociation opacity (see Chubb et al. 2024a, Sect. 8 for a discussion on currently-available UV opacities and planned advances).

An additional challenge, which will also be relevant for the ELTs, is that HWO and Roman will enable reflection spectra, in some cases phase-resolved for HWO, to be obtained for a much wider range of planets. This will require accurate multiple scattering models, which are slower to run than models operating under an extinction-only approximation (e.g. Lupu et al. 2016; Damiano and Hu 2020). It may also be necessary to include the effects of polarization – either to model the scattering of light by clouds or the reflection by surface liquids on terrestrial planets (e.g., Gordon et al. 2023; Vaughan et al. 2023). At the time of writing, whilst polarized light models exist for exoplanet atmospheres (e.g. Chubb et al. 2024b), these are yet to be included in a retrieval context. There will be an additional forward modelling burden to account for this, and also larger datasets if polarized light measurements are combined with total flux in a retrieval; however, extrapolating from other instances where datasets containing different information about the same target are combined (for example, the joint high- and low-resolution retrievals described above), the additional constraints that could be obtained, particularly for cloud species, are likely to be worth the time investment.

Finally, the European Space Agency identified in their Voyage 2050 prioritization exercise that one of the next L-class missions should be a far-infrared telescope, with the primary aim of characterizing habitable zone exoplanets. One mission concept likely to be proposed for this is the Large Interferometer For Exoplanets (LIFE; Quanz et al. 2022). Many of the challenges for interpreting spectra from LIFE would be the same as for any direct imaging observations, but the wavelength range planned for LIFE will overlap with that of JWST. A key consideration will be the provision of infrared opacity data for complex molecules that could be found in cool and temperate atmospheres.

### Outlook and summary

Spectral retrieval remains a powerful computational tool for spectroscopic analysis of transiting exoplanets and directly imaged companions. The parametric nature and flexibility of retrieval models result in a unique ability to fit for parameters beyond constraints imposed by current understanding of chemistry, structure and dynamics whilst at the same time providing robust statistical analysis, allowing for observationally informed development in our knowledge of planetary atmospheric physics. As the information content of available data has exponentially increased in recent years, so too has the number of model parameters required and the associated computational burden.

Here, we briefly summarize the main findings of this review. We hope this summary can also provide some recommendations for exoplanet scientists wishing to experiment with spectral retrieval for the first time.

**Beware the black box:** Setting out to perform a retrieval requires decisions to be made, e.g. about priors, the likelihood function, and which parameters to vary. We have demonstrated that all of these choices impact the eventual result of the retrieval, and in particular that using Bayesian evidence comparisons between models to determine detection significance is fraught with problems in cases where the models being compared are incomplete. In the landscape of new and improved data, retrieval standards also need to evolve, so using an ‘out of the box’ retrieval setup that worked for one dataset is not guaranteed to be adequate for a different target or instrument.

**Computational efficiency:** As retrieval models become increasingly complex, the number of CPU hours invested increases drastically. Where the number of free parameters cannot be reduced without compromising the retrieval result, the limiting factor for the retrieval runtime is the forward model calculation and its efficiency.

Generally speaking, astronomers are not trained as software engineers, and whilst we as a field are capable of writing code that solves complex physical problems, we do not necessarily do so in the most efficient way. In an ideal world, retrieval codes should all benefit from the expertise of a software engineer to ensure efficiency and code stability. In practice, funding considerations do not usually allow for this, with the result that most codes are likely to run less efficiently than they could or should. We would advocate for funding opportunities that acknowledge this challenge, and more readily allow for employing software engineers to work on scientific code.

Machine learning approaches, which are on the rise, will also change the computational landscape in the future. Whilst training a network can be computationally intensive, evaluation is very efficient and can speed up the retrieval process. This situation is rapidly evolving and we will update the status of machine learning retrievals in future work.

**Future missions:** Future missions will inevitably place yet more pressure on the retrieval process, with the requirement for more complex forward models (e.g. multiple scattering retrievals for reflected light spectroscopy with HWO) and larger data volumes (e.g. Ariel). The dramatic shift in required model complexity and fidelity at the start of JWST has been a useful learning experience for rapid adaptation of retrieval models to new scenarios. Forward planning for these missions will be key, since especially for the ELTs and HWO these are likely to involve substantial changes in existing model frameworks to make them suitable for incoming data.

### Final conclusions

Going into the next decades of exoplanet exploration, a key focus for retrievals must be improving efficiency, either in the basics of the forward model calculation, the overall code architecture, or parameterization choices. This is an environmental consideration as well as a practical one. Appropriate forward planning for a retrieval analysis can substantially reduce the number of computations required to arrive at a robust solution. In an era where the majority of spectral retrieval codes for exoplanets are (rightly) open-source, we stress that use of these codes as a black box without any understanding of the underlying physics and mechanics is likely to result not only in poor quality analyses, but also a waste of computational resource. Productive collaboration and mentorship is a must for the spectral retrieval community, and we particularly recommend newcomers to take advantage of summer schools and hackathons, which often include code tutorials.

## Data Availability

No datasets were generated or analysed during the current study.

## Footnotes

1 https://roman.gsfc.nasa.gov/images/pdf/Roman-Instruments-Chart.pdf
